# A Mechanistic Pharmacokinetic/Pharmacodynamic Model for Sequence-Dependent Synergy in Pemetrexed–Osimertinib Combinations Against Non-Small Cell Lung Cancer (NSCLC): Translational Insights

**DOI:** 10.3390/pharmaceutics18040408

**Published:** 2026-03-26

**Authors:** Kuan Hu, Yan Lin, Huachun Ji, Tong Yuan, Yu Xia, Jin Yang

**Affiliations:** Center of Drug Metabolism and Pharmacokinetics, China Pharmaceutical University, Nanjing 211198, China; 3119010074@stu.cpu.edu.cn (K.H.);

**Keywords:** pemetrexed, osimertinib, synergy effect, schedule-dependent, pharmacokinetic/pharmacodynamic (PK/PD) modeling

## Abstract

**Background and Objectives:** Combining osimertinib (OSI) with pemetrexed (PEM) can enhance antitumor efficacy; however, the benefit is schedule-dependent. Our previous pharmacodynamic (PD) study showed that concurrent PEM + OSI is limited by OSI-induced G1 arrest, attenuating early PEM cytotoxicity. In contrast, sequential PEM→OSI allows PEM to fully induce S-phase arrest and DNA damage but elicits a pro-survival EGFR rebound; subsequent OSI suppresses this rebound and promotes apoptosis of damaged cells, yielding strong synergy. Here, we investigated whether pharmacokinetic (PK) drug–drug interactions (DDIs) contribute to this synergy and predicted the relative advantage of PEM→OSI versus PEM + OSI under clinically relevant conditions using a PK/PD approach. **Method and Results:** Potential PK-DDIs were assessed at cellular uptake, plasma exposure, and intratumoral distribution levels. No meaningful PK-DDIs were observed, supporting a primary PD-driven synergy. We integrated mouse PK with in vitro/in vivo PD data to build a mechanistic Quantitative System Pharmacology (QSP)–PK–PD model linking drug disposition to folate biology, Epidermal Growth Factor Receptor (EGFR) signaling, and tumor growth inhibition. The model recapitulated schedule dependence and explained PEM→OSI superiority: PEM initiates damage and EGFR compensatory rebound, after which OSI suppresses EGFR signaling and enhances apoptosis. In contrast, concurrent PEM + OSI induced G1 arrest, reduced the pool of damaged apoptosis-susceptible cells, and weakened the synergy. Global sensitivity analysis identified intrinsic OSI sensitivity and the pro-apoptotic protein Bim as key determinants; reduced OSI sensitivity or Bim activity diminished the advantage of the sequential strategy. The simulations indicated that OSI can start 48 h after PEM exposure (no extended drug holiday is needed) and that the PEM→OSI benefit remains robust across heterogeneity, including BIM-deletion polymorphisms and inter-individual variability in tumor drug sensitivity. **Conclusions:** This mechanism-based QSP–PK–PD framework connects whole-body PK to core PD processes, explains schedule-dependent synergy, and supports optimization of sequencing intervals and identification of likely responders.

## 1. Introduction

Primary lung cancer is one of the most common and deadly malignancies, with NSCLC accounting for approximately 80% of all cases. Epidermal Growth Factor Receptor (EGFR) mutations are present in more than 50% of Asian and 10–20% of European NSCLC patients [1,2]. EGFR tyrosine kinase inhibitors (EGFR-TKIs) markedly suppress the proliferation of EGFR-mutant NSCLC cells and induce apoptosis, and they have become a cornerstone of therapy for EGFR-mutant NSCLC [3]. Although the third-generation EGFR-TKI osimertinib (OSI) has demonstrated robust first-line efficacy in the AURA3 [4] and FLAURA [5] clinical trials, most patients ultimately develop disease progression [5]. Therefore, combination strategies have been explored to mitigate resistance associated with single-target inhibition [6]. The NEJ009 [7] and JMIT [8] trials showed that gefitinib plus chemotherapy improved both progression-free survival (PFS) and overall survival (OS). This synergy may arise from the non-target-specific cytotoxic effects of chemotherapy, which can partially offset tumor heterogeneity, delay the emergence of EGFR-TKI resistance, and suppress epithelial–mesenchymal transition [9,10,11]. Consistently, in the FLAURA2 study [5], OSI combined with a pemetrexed (PEM)-based platinum doublet for four cycles of induction therapy, followed by maintenance OSI plus PEM until progression, achieved a significant PFS benefit at interim analysis. Although OS data remained immature, a favorable trend was observed.

Despite these benefits, concurrent administration may not be optimal because the synergistic effect of chemotherapy and EGFR-TKIs can depend on the dosing order and timing [12,13,14,15]. A sequential strategy (PEM→EGFR-TKI) may optimize the dosing order to avoid antagonism between chemotherapy and molecular targeted therapy caused by cell-cycle interference [12,13,14,15] while leveraging EGFR-TKI-mediated chemo-sensitization to achieve stronger synergy [3,12,13,14,15]. Recently, sequential PEM→Aumolertinib achieved an objective response rate (ORR) of up to 93.3% [3], highlighting the clinical potential of the sequential strategy for third-generation EGFR-TKIs.

**Figure 1:**
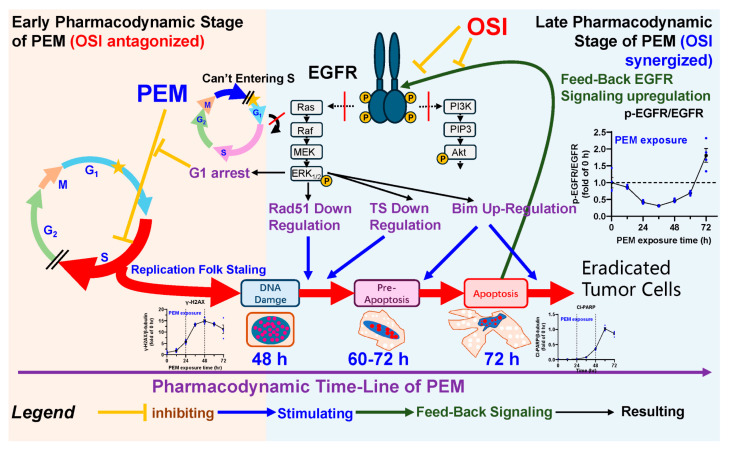
**Schematic diagram of the sequence-dependent synergistic mechanism between PEM and OSI** [12]. We divided the pharmacodynamic process of PEM into two stages: the early stage, characterized by S-phase arrest and DNA damage accumulation, and the late stage, marked by peak DNA damage, initiation of apoptotic signaling, cellular apoptosis, and feedback EGFR signal rebound. Sufficient accumulation of early-stage effects is a prerequisite for initiating the late-stage effects. OSI exerts potent antagonistic effects by counteracting PEM-induced cytotoxicity through G1-phase arrest during the early stage, as G1-arrested tumor cells cannot enter S phase. However, once PEM’s pharmacodynamic process progresses to the late stage, OSI synergizes with PEM’s late-phase effects through multiple mechanisms: downregulating Rad51 to impair DNA damage repair, suppressing PEM sensitivity marker thymidylate synthase (TS), upregulating pro-apoptotic protein Bim, and directly inhibiting feedback EGFR signaling pathways, thereby promoting PEM-induced pre-apoptotic signaling and exerting chemo-sensitizing effects. The accumulation of PEM’s early-stage effects requires approximately 48 h for completion. Premature OSI exposure before this timeframe will antagonize the accumulation of PEM’s early effects. Consequently, the PEM + OSI concurrent strategy or 24 h interval PEM→OSI strategy fails to circumvent early-stage antagonism, thereby preventing achievement of elevated DNA damage and apoptotic signaling status required for OSI-mediated synergy. In contrast, the 48 h interval sequential PEM→OSI regimen simultaneously circumvents OSI’s antagonism of early-phase PEM effects and fully leverages OSI’s potentiation of late-phase effects, demonstrating superior therapeutic potential.

In our previous work [12], we explored the preclinical pharmacodynamic basis for the schedule-dependent synergy between PEM and OSI. Specifically, we demonstrated that sequential PEM→OSI was superior to concurrent PEM + OSI and proposed a mechanistic hypothesis explaining this advantage in terms of avoidance of early-stage antagonism and enhancement of late-stage synergistic effects, as summarized in Figure 1. This previous study, therefore, provided a PD-level rationale for selecting the PEM→OSI regimen with a 48 h interval. However, several key questions remain unresolved. It is still unclear whether the observed synergy is driven solely by PD interactions or is also influenced by pharmacokinetic (PK) interactions. In addition, although our previous study clarified the mechanistic basis of schedule dependence at the preclinical level, it did not provide a quantitative framework for translating these findings into clinically relevant predictions or for informing subsequent clinical investigation.

In addition to PD interactions, PK interactions may also contribute to chemotherapy–molecular-targeted therapy synergy by altering systemic exposure or intratumoral drug levels [3,16]. Clinically, PEM is administered on a 21-day cycle (q21d), but its plasma concentration remains above therapeutically effective levels for only a short window of up to 48 h [17]. Consequently, efficient tumor drug uptake during this transient period of high systemic exposure is a critical determinant of antitumor activity [3,18], as intracellularly trapped PEM polyglutamates are responsible for its sustained cytotoxic effect [19]. Intracellular PEM accumulation depends on pH-sensitive transport, including reduced folate carrier (RFC) and proton-coupled folate transporter (PCFT)-mediated uptake and breast cancer resistance protein (BCRP)-mediated efflux, which increases as the pH of the tumor microenvironment approaches neutrality [18,20]. Because OSI competitively inhibits BCRP under mildly acidic conditions [18,20,21], it may promote vascular normalization [3,22] and modulate tumor endothelial cell lactate secretion [22], thereby it may increase intratumoral PEM exposure by improving delivery, shifting microenvironmental pH toward neutrality, and reducing efflux by inhibiting BCRP. However, the PK interactions between PEM and OSI remain unclear.

From a translational perspective, although FLAURA2 has established the benefit of the PEM + OSI combination regimen [5], the highly promising sequential PEM→OSI strategy [3] still lacks validation in large-scale clinical trials. In this context, it is crucial to (1) predict the potential clinical advantage of the sequential PEM→OSI strategy over concurrent PEM + OSI across scenarios, (2) identify the key biological and pharmacological determinants of synergistic efficacy, and (3) characterize the patient subpopulations most likely to benefit. Mathematical modeling [23] and quantitative pharmacology PK/PD approaches [24] provide effective frameworks for integrating preclinical evidence to support such predictions. Several “top-down” descriptive synergy models have been developed for sequential combination therapies [16,25,26,27,28,29,30]. However, these descriptive models are structurally simple. They merely quantified the fold-change in a drug’s PD parameters (increase for synergy and decrease for antagonism) under a given combination strategy, without mathematically encapsulating the pharmacodynamic interactions between the two drugs. Consequently, they are limited to describing the dataset used for modeling and lack the ability to extrapolate to untested scenarios or identify key determinants; these shortcomings limit their translational potential.

As illustrated in Figure 1, the schedule-dependent synergy of the PEM–OSI combination arises from time-varying interactions across multiple processes, including cell cycle regulation, DNA damage induction and repair, apoptotic signaling, and EGFR pathway dynamics, forming a complex dynamic network [12]. The “bottom-up” Quantitative System Pharmacology (QSP)–PK/PD approach [28,29,31,32,33,34], which integrates drug mechanisms, signaling networks, disease biology, and whole-body physiology, is therefore particularly well suited for characterizing the sequence-dependent synergy between PEM and OSI and for capturing such time-dependent interactions. This QSP framework allows synergy to emerge from the mechanistic structure of the model and could help identify the key determinants of antitumor efficacy.

**Figure 2:**
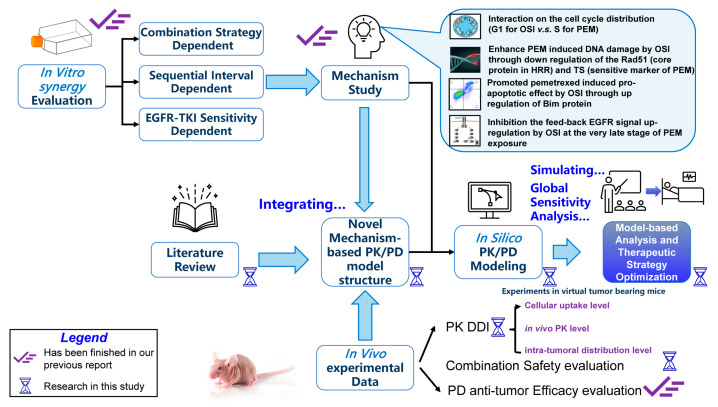
**Overall research strategy of the present study**. We first evaluated the PK-DDI potential between PEM and OSI. We then integrated in vitro and in vivo PK/PD data, mechanistic insights into sequence-dependent synergy, and relevant literature information to develop a mechanistic QSP-PK-PD model. This model was used to explore key factors influencing synergistic efficacy, to optimize the sequential dosing interval, and to compare the relative advantages of PEM→OSI versus PEM + OSI under complex clinical scenarios. Purple check marks indicate tasks completed in our previous work [12], whereas the blue hourglass represents work conducted in the current study.

Compared with our previous work [12], which focused on the pharmacodynamic basis of schedule-dependent PEM–OSI synergy, the present study was designed to extend the mechanistic understanding of PEM–OSI synergy into a translational quantitative framework. As illustrated in Figure 2, we first evaluated whether PK-DDI between PEM and OSI contributes to the observed synergy at the levels of cellular drug uptake, systemic plasma pharmacokinetics, and intratumoral distribution. We then integrated these PK data with in vitro and in vivo PD results, together with our prior mechanistic understanding [12], to develop a mechanistic QSP–PK–PD model. Finally, we used this model to simulate and compare sequential PEM→OSI and concurrent PEM + OSI regimens across multiple clinically relevant scenarios, with aims of optimizing the sequential interval, identifying the key biological and pharmacological determinants of synergy, and characterizing the patient subpopulations most likely to benefit from the sequential strategy.

## 2. Materials and Methods

### 2.1. Pharmacokinetic Drug–Drug Interaction (PK-DDI) Studies

All drugs, reagents, cell lines, and animals used in this study are commercially available, and their catalog numbers and manufacturers are listed in Table S1 and Figures S8–S10. All in vivo experiments in this study strictly adhered to protocols approved by the Animal Ethics Committee of China Pharmaceutical University (Approval No. YSL-202504062). Detailed procedures for cell culture, tumor inoculation, and Liquid Chromatography–Tandem Mass Spectrometry (LC–MS/MS) bioanalysis for quantifying PEM and OSI in biological matrices are provided in Supplementary Methods S1.1–S1.3, Tables S3–S13, and Figures S1 and S2; notably, the assay only requires 10 μL of plasma. The PK-DDI study comprised three components: cellular uptake, in vivo pharmacokinetics, and intratumoral distribution. The rationale for dose and concentration selection for each PK-DDI experiment is provided in Supplementary Method S1.4. [3,11,13,17,35,36,37]

#### 2.1.1. PK-DDI Study at the Cellular Uptake Level

Four EGFR-mutation-positive NSCLC cell lines, HCC827, PC9, NCI-H1975, and NCI-H1650 cells (their genetic characters were summarized at Table S2 [12,38,39,40,41,42]), were seeded in 24-well plates at densities of 3.0 × 10^5^, 1.5 × 10^5^, 1.5 × 10^5^, and 1.5 × 10^5^ cells in 1 mL of RPMI1640 (containing 10% fetal bovine serum, FBS) per well, respectively. After allowing the cells to adhere, they were rinsed once with 1 mL of warm PBS. The culture medium was replaced with 1 mL of serum-free RPMI1640 medium containing OSI (1 μM) or PEM (100 μM) for drug administration, and the administration time was immediately recorded. At harvest, the cells were washed three times with 1 mL of ice-cold PBS, followed by the addition of 200 μL of ultrapure water. The plates were transferred to a −80 °C freezer and subjected to three freeze-thaw cycles to ensure complete lysis. The cells were subsequently scraped, and the lysates were collected and sonicated using an ultrasonic cell disruptor (SCIENTZ08-II, SCIENTZ, Ningbo, China). Protein concentrations in the cell lysates were determined using the BCA assay. LC–MS/MS-quantified drug concentrations were then normalized to protein concentration using the equation $C_{\mathrm{normalized},\mathrm{lysate}}=C_{\mathrm{drug},\mathrm{lysate}}/C_{\mathrm{protein},\mathrm{lysate}}$.

#### 2.1.2. In Vivo Pharmacokinetic Drug–Drug Interaction Research

In the in vivo PK study, PEM was administered via intraperitoneal injection in saline (intravenously for rats), whereas OSI was delivered via oral gavage in 1% Tween-80. Blood sampling from both rats and mice was performed via the retro-orbital venous plexus. To minimize blood loss in the mice, each dosing cohort (*n* = 15; monotherapy or concurrent dosing) was divided into three subgroups (*n* = 5). Subgroups were alternated for serial blood sampling, with each mouse bled at 3-4 time points (approximately 50 μL of whole blood per draw). Following centrifugation, approximately 30 μL of plasma was obtained, which was sufficient for LC-MS/MS analysis (requiring 10 μL). The detailed plasma sampling schedules are listed in Table S13. In contrast to the rats, from which individual PK profiles were derived, mouse data within each cohort were pooled to generate a single mean concentration–time curve with error bars.

To investigate the potential PK-DDI between PEM and OSI following co-administration, 18 male Sprague-Dawley (SD) rats (approximately 200 g) were randomly assigned to three groups: PEM monotherapy, OSI monotherapy, and the combination group (PEM + OSI). The doses were 70 mg/kg for PEM and 10 mg/kg for OSI. Plasma concentration–time profiles were plotted, and non-compartmental analysis (NCA) parameters were calculated using WinNonlin and are summarized in Tables S14 and S15.

Clinically, PEM is administered on a 21-day cycle (q21d), whereas OSI is administered once daily (q1d). To examine whether chronic OSI administration alters the PK of PEM, a study was performed using 12 male SD rats (~200 g). Rats were randomized into control and OSI-pretreated groups. The control group received vehicle (1% Tween-80) daily for eight days, whereas the OSI pretreated group received OSI (10 mg/kg) daily for eight days. On day 8, both groups were administered PEM (70 mg/kg). Plasma concentration–time profiles of PEM were established, and the NCA parameters were calculated and are summarized in Table S16.

Finally, the potential PK-DDI between PEM and OSI was evaluated in two distinct tumor-bearing mouse models: NCI-H1975-bearing NOD-SCID mice and PC9-bearing Balb/c nude mice. Studies were initiated when tumor volumes reached approximately 1000 mm^3^, with doses of 100 mg/kg for PEM and 5 mg/kg for OSI, respectively. The objectives were (1) to validate the PK-DDI findings in a realistic tumor-bearing model that incorporates disease-related factors and (2) to provide PK parameters for subsequent modeling studies. The experimental design in tumor-bearing mice was similar to that in rats; however, owing to the limited number of tolerated blood sampling points in mice, a single sampling schedule could not adequately capture the PK profiles of both drugs. Therefore, the DDI assessment was conducted in two separate arms: one comparing the PK of PEM under monotherapy versus concurrent PEM + OSI dosing and the other comparing the PK of OSI under monotherapy versus concurrent PEM + OSI dosing. Because individual NCA parameters could not be derived, NCA analysis of the mice was performed using the mean concentration–time curve.

A significant PK-DDI was considered absent if the ratio of parameters (concurrent/monotherapy) was within the range of 0.8–1.25.

#### 2.1.3. Effect of Long-Term OSI Treatment on the Intra-Tumoral Distribution of PEM in Tumor-Bearing Mice

To investigate whether OSI could influence intratumoral exposure to PEM via vascular normalization, we conducted an experiment based on the study by Ao et al. [3]. As illustrated in Figure 5H, NOD-SCID mice bearing NCI-H1975 tumors (approximately 800 mm^3^ in volume) were divided into two groups: the control group and the OSI 8-day group. The control group received 1% Tween-80 continuously for eight days, while the OSI group received OSI (5 mg/kg) continuously for eight days. On day 8, both groups were simultaneously administered PEM (100 mg/kg). Four hours after PEM administration, blood was collected via retro-orbital puncture to obtain plasma, and tumor tissues were harvested. Tumor tissues were added to 1× PBS and homogenized under freezing conditions at a weight-to-volume ratio of 1:3 to 1:5 (*w*/*v*). The concentration of PEM in plasma and tumor tissue homogenates was determined using the LC-MS/MS method.

### 2.2. In Vivo Anti-Cancer Efficacy and Safety Evaluation

This section presents data from our previous study [12] evaluating the in vivo antitumor efficacy of PEM and OSI administered as monotherapies or in combination under different treatment strategies. Briefly, female 7-week-old BALB/c nude mice with an average body weight of approximately 20 g were used to establish tumor xenografts. HCC827 cells (1 × 10^7^ cells per mouse), suspended in 50% high-concentration Matrigel, were subcutaneously inoculated into the right flank of the mice. Once the tumor volumes reached approximately 200 mm^3^, the animals were randomly assigned to treatment groups and treated according to the dosing schedule shown in Figure 6A. Tumor size and body weight were monitored every 3 days. For tumor volume assessment, the longest diameter (a) and shortest diameter (b) were measured using electronic calipers (Mitutoyo, Kanagawa, Japan), and the tumor volume was calculated as $V_{\mathrm{tumor}}=\left(\pi /6\right)\times ab^{2}$ [43]. Hepatic and renal toxicity were evaluated by morphological examination of H&E-stained liver and kidney sections.

PEM was administered intraperitoneally (i.p.) at 35 mg/kg three times daily (t.i.d.) with a 4 h dosing interval. This regimen was selected because PEM has a short elimination half-life in mice (~35 min), and our previous study showed that approximately 48 h of continuous exposure is required for robust cytotoxic activity [12]. This schedule yielded a total daily dose of 105 mg/kg, which is comparable to doses used in related studies (~100 mg/kg) [3,9,11,13], while providing more sustained PEM exposure. OSI was administered at a dose of 1 mg/kg because HCC827 tumor cells are relatively sensitive to OSI.

### 2.3. Development of a Mechanistic QSP–PK–PD Model for Sequence-Dependent Effects of PEM-OSI Combination Therapy

#### 2.3.1. Overall Model Structure

**Figure 3:**
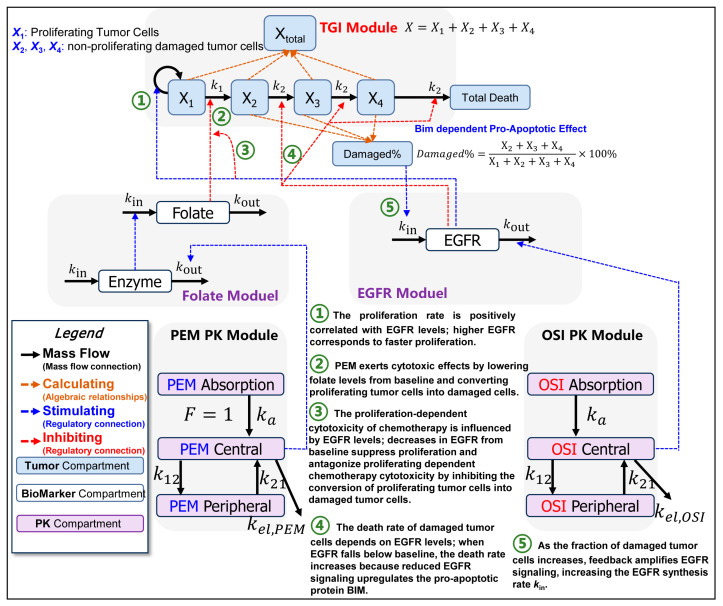
**Schematic representation of the QSP-PK-PD model to describe the sequence-dependent synergy effect of PEM-OSI combined therapy.** This model was developed based on our pharmacological knowledge [12] of intrinsic mechanisms within the PEM-OSI combination and could simulate all groups (monotherapy and combination therapies with different strategies) using the same model structure and the same set of model parameters. The model consists of five major modules: PEM PK module, OSI PK module, folate module, EGFR module, and tumor growth inhibition (TGI) module. Interaction effects exist between the modules.

By integrating PK data (PC9-BALB/c nude in Figure 5D) with in vitro (Figure 7) and in vivo PD data (Figure 6A,B), as well as our previous understanding of the pharmacological mechanism [12] (Figure 1), we developed a QSP–PK–PD model, which is presented in Figure 3. In the figure, the solid lines denote mass–balance connections, dashed lines denote regulatory links, calculation nodes (orange) indicate algebraic relationships (e.g., defining compartment masses such as total tumor volume), and stimulation/inhibition (blue/red) symbols indicate the modulation of specific reaction rates. The model comprises five modules: PEM-PK, OSI-PK, Folate, EGFR, and tumor growth inhibition (TGI). The EGFR module interacts with the TGI module.

#### 2.3.2. PEM and OSI PK Module

**Figure 4:**
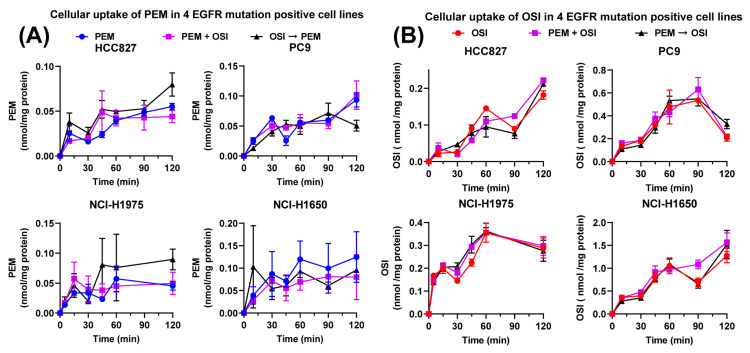
**Cellular uptake of PEM and OSI in 4 EGFR-positive NSCLC cell lines under different treatment conditions** (*n* = 4). Panel (**A**) shows the uptake of PEM, while panel (**B**) shows the uptake of OSI. The drug concentrations in the medium were 100 μM for PEM and 1 μM for OSI. The experimental scenarios were defined as follows: “PEM”: cells exposed to PEM alone; “OSI”: cells exposed to OSI alone; “PEM + OSI”: cells exposed to both drugs simultaneously; “PEM → OSI”: cells pretreated with 100 μM PEM for 8 h prior to OSI uptake; “OSI → PEM”: cells pretreated with 1 μM OSI for 8 h prior to PEM uptake. All data are presented as mean ± SD.

**Figure 5:**
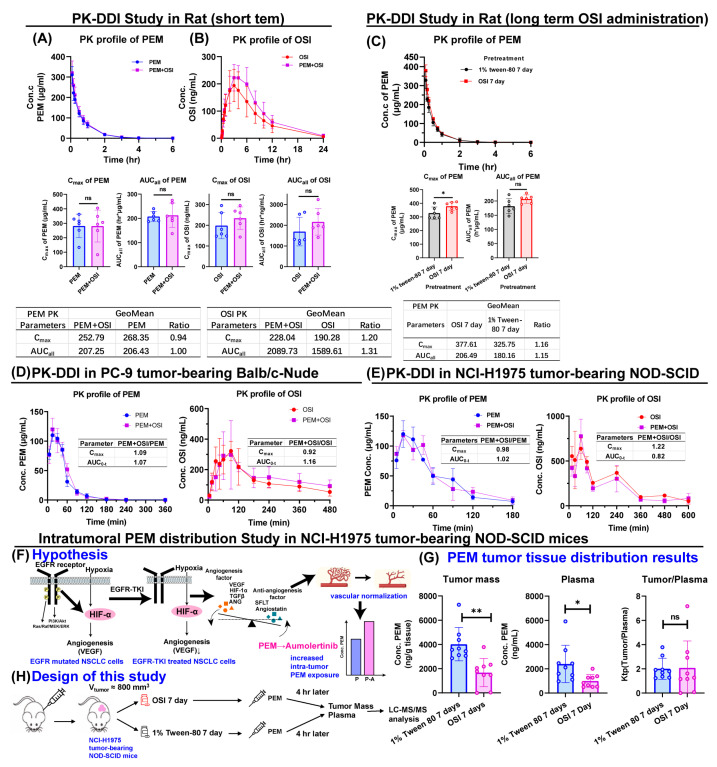
**Lack of PK-DDI both at the systemic plasma concentration level (rat and tumor xenograft mice, (A–E)) and tumor tissue distribution level (F,G).** (**A**,**B**): Concurrent administration of PEM and OSI did not result in PK-DDI in rats (*n* = 6). (**C**): Prior administration of OSI for 7 consecutive days did not alter the PK profile of PEM in rats (*n* = 6). (**D,E**): Similarly, no PK-DDI was observed in PC-9-Balb/c nude mouse xenografts (**D**) or NCI-H1975-NOD-SCID mouse xenografts (**E**) following concurrent administration of both drugs (*n* = 5). (**F**): Schematic illustrating the potential effect of EGFR-TKI-induced vascular normalization on the intratumoral distribution of PEM [3]. (**G**): Although OSI pretreatment for 7 days reduced PEM concentrations in both tumor and plasma, the tumor-to-plasma partition coefficient *K*_tp_ remained unchanged, suggesting that OSI does not significantly affect the intratumoral distribution of PEM (*n* = 9). (**H**) Study design for intratumoral PEM distribution research. All data are presented as mean ± SD; *ns*: not significant; * *p* < 0.05; ** *p* < 0.01.

Based on population PK reports, both PEM [17,44,45,46,47] and OSI [21,35,48,49,50,51] were characterized using a two-compartment model. Because no meaningful PK-DDI was detected between PEM and OSI at any level (Figure 4 and Figure 5), the PK profiles under the combination scenario were simulated using the PK parameter sets derived from monotherapy (Figure S4A,B).

As illustrated in Figure 3 and Figure S4A, the PK behavior of PEM is described using differential Equations (1)–(3). PK parameters were obtained from WinNonlin fitting, assuming that PEM is not metabolized and administered via intraperitoneal injection with bioavailability fixed at 1: $C_{\mathrm{pem},1}=X_{\mathrm{pem},1}/V_{1,\mathrm{pem}}$.

(1) $$\frac{dX_{\mathrm{pem},a}}{dt}=-k_{a,\mathrm{pem}}X_{\mathrm{pem},a}$$

(2) $$\frac{dX_{\mathrm{pem},1}}{dt}=k_{a,\mathrm{pem}}X_{\mathrm{pem},a}-k_{12,\mathrm{pem}}X_{\mathrm{pem},1}+k_{21,\mathrm{pem}}X_{\mathrm{pem},2}-k_{\mathrm{el},\mathrm{pem}}X_{\mathrm{pem},1}$$

(3) $$\frac{dX_{\mathrm{pem},2}}{dt}=k_{12,\mathrm{pem}}X_{\mathrm{pem},1}-k_{21,\mathrm{pem}}X_{\mathrm{pem},2}$$

As shown in Figure 3 and Figure S4B, the PK behavior of OSI is described using differential Equations (4)–(6). The model parameters were obtained via WinNonlin fitting: $C_{\mathrm{osi},1}=X_{\mathrm{osi},1}/{(V}_{\mathrm{osi},1}/F_{a,\mathrm{osi}})$.

(4) $$\frac{dX_{\mathrm{osi},a}}{dt}=-{F_{a,\mathrm{osi}}k}_{a,\mathrm{osi}}X_{\mathrm{osi},a}$$

(5) $$\frac{dX_{\mathrm{osi},1}}{dt}={F_{a,\mathrm{osi}}k}_{a,\mathrm{osi}}X_{\mathrm{osi},a}-k_{12,\mathrm{osi}}X_{\mathrm{osi},1}+k_{21,\mathrm{osi}}X_{\mathrm{osi},2}-k_{\mathrm{el},\mathrm{osi}}X_{\mathrm{osi},1}$$

(6) $$\frac{dX_{\mathrm{osi},2}}{dt}=k_{12,\mathrm{osi}}X_{\mathrm{osi},1}-k_{21,\mathrm{osi}}X_{\mathrm{osi},2}$$

#### 2.3.3. Folate and EGFR Signaling Module

The folate module describes PEM-induced perturbations in folate metabolism using differential Equations (7) and (8), where *Enzyme* and *Folate* represent the normalized levels of folate-metabolizing enzymes and folate, respectively. *E*_max,pem_ and *EC*_50,pem_ are PD parameters that characterize the PEM-driven changes in folate levels.

(7) $$\frac{dEnzyme}{dt}=k_{\mathrm{out},\mathrm{enzyme}}-k_{\mathrm{out},\mathrm{enzyme}}\cdot Enzyme\cdot (1+\frac{E_{\max,\mathrm{pem}}C_{\mathrm{pem}}}{EC_{50,\mathrm{pem}}+C_{\mathrm{pem}}})$$

(8) $$\frac{dFolate}{dt}=Enzyme^{\gamma_{Enzyme}}k_{\mathrm{out},\mathrm{folate}}-k_{\mathrm{out},\mathrm{folate}}\cdot Folate$$

Via differential Equation (9), The EGFR module describes the effects of OSI on the EGFR signaling pathway and the process of EGFR signal rebound driven by damaged tumor cells [12,13,52] (Figure 1 and Figure 3). EGFR denotes normalized EGFR levels. EGFR and downstream signaling rebound at the late stages of PEM exposure have been confirmed in our previous study [12] and in other reports [52]. In this study, we mathematically reproduced this process by increasing the cell-damage-dependent synthesis of EGFR (*k*_in_) (Figure 1 and Figure 3, Equation (9)). As an irreversible inhibitor, OSI binds to EGFR receptors and depletes the available receptor pool. NSCLC can restore EGFR signaling only by resynthesizing EGFR receptors [53]. The EGFR signal turnover rate $k_{\mathrm{out},\mathrm{EGFR}}$ was estimated from in vitro experiments (Figure 7E,F). OSI drives EGFR inactivation through PD parameters, including $I_{\max,\mathrm{osi}}$, $EC_{50,\mathrm{osi}}$, and $\gamma_{\mathrm{osi}}$.

(9) $$\frac{dEGFR}{dt}=k_{\mathrm{out},\mathrm{EGFR}}\left(1+k_{\mathrm{feedback}}\cdot Damaged{\%}^{\gamma_{\mathrm{feedback}}}\right)-k_{\mathrm{out},\mathrm{EGFR}}\cdot EGFR\cdot (1+\frac{{I_{\max,\mathrm{osi}}\cdot C}_{osi}^{\gamma_{osi}}}{EC_{50,\mathrm{osi}}^{\gamma_{osi}}+C_{osi}^{\gamma_{osi}}})$$

Here, *Damaged*% denotes the proportion of damaged tumor cells in the total tumor cell population (Equation (15)), and *k*_feedback_ and $\gamma_{\mathrm{feedback}}$ are PD parameters that characterize the damaged tumor cell-driven EGFR signaling feedback rebound.

#### 2.3.4. Tumor Growth Inhibition (TGI) Module and Its Interaction with EGFR Signaling

The tumor growth-inhibition (TGI) module was constructed based on the Simeoni model [54] and integrates folate levels, EGFR signaling, and their interactions with tumor dynamics. The tumor was represented by four compartments: proliferating tumor cells (X_1_) and tumor cells in a series of sequentially damaged states (X_2_, X_3_, and X_4_). The total tumor volume was defined as $X=X_{1}+X_{2}+X_{3}+X_{4}$. Parameter $k_{1}$ denotes the natural transition rate constant from proliferating to damaged cells in the absence of drug intervention, whereas $k_{2}$ denotes the natural apoptosis rate constant of damaged tumor cells in the absence of drug intervention. The TGI module is described by the differential Equations (10)–(17) as follows:

(10) $$\frac{dX_{1}}{dt}=\frac{\lambda_{0}X_{1}}{{\left[1+{\left(\frac{\lambda_{0}}{\lambda_{1}}X_{1}\right)}^{\psi }\right]}^{\frac{1}{\psi }}}EGFR^{\gamma_{\mathrm{EGFR}}}-k_{1}X_{1}\left(1+\frac{E_{\max,\mathrm{folate}}{\left(1-folate\right)}^{\gamma_{\mathrm{folate}}}}{EC_{50,\mathrm{folate}}^{\gamma_{\mathrm{folate}}}+{\left(1-folate\right)}^{\gamma_{\mathrm{folate}}}}\right)\cdot EGFR^{\gamma_{G1}}$$

(11) $$\frac{dX_{2}}{dt}=k_{1}X_{1}\left(1+\frac{E_{\max,\mathrm{folate}}\left(1-folate\right)}{EC_{50,\mathrm{folate}}+\left(1-folate\right)}\right)\cdot EGFR^{\gamma_{G1}}-k_{2}X_{2}\left(1+k_{\mathrm{bim}}{\left(1-EGFR\right)}^{\gamma_{\mathrm{bim}}}\right)$$

(12) $$\frac{dX_{3}}{dt}=k_{2}X_{2}\left(1+k_{bim}{\left(1-EGFR\right)}^{\gamma_{bim}}\right)-k_{2}X_{3}\left(1+k_{bim}{\left(1-EGFR\right)}^{\gamma_{bim}}\right)$$

(13) $$\frac{dX_{4}}{dt}=k_{2}X_{3}\left(1+k_{bim}{\left(1-EGFR\right)}^{\gamma_{bim}}\right)-k_{2}X_{4}\left(1+k_{bim}{\left(1-EGFR\right)}^{\gamma_{bim}}\right)$$

(14) $$X=X_{1}+X_{2}+X_{3}+X_{4}$$

(15) $$Damaged\%=\left(X_{2}+X_{3}+X_{4}\right)/\left(X_{1}+X_{2}+X_{3}+X_{4}\right)\times 100\%$$

(16) $$X1\%={(X}_{1}/(X_{1}+X_{2}+X_{3}+X_{4}))\times 100\%\;$$

(17) $$Total\;Dead=\int_{0}^{\infty }k_{2}X_{4}\left(1+k_{bim}{\left(1-EGFR\right)}^{\gamma_{bim}}\right)$$

PEM-induced cytotoxicity occurs when folate levels decrease below baseline levels. Pharmacologically, PEM sequentially induces S-phase arrest, DNA damage, and pro-apoptotic signaling (Figure 1 and Figure 3) [55]. In this model, this process is expressed mathematically as a PEM driving proliferating tumor cells in compartment X_1_ to transit into non-proliferating, damaged tumor cells in compartment X_2_ (Figure 3). This process is described by Equations (10) and (11) and is governed by PD parameters, such as $E_{\max,\mathrm{folate}}$ and $EC_{50,\mathrm{folate}}$. In addition, an increased proportion of damaged cells also leads to a rebound in EGFR signaling, thereby hindering the cytotoxicity of PEM (Equation (9), Figure 1 and Figure 3).

The OSI-induced decrease in EGFR levels from baseline exerts four major effects: (1) an anti-proliferative effect that directly reduces the proliferation rate of the X_1_ compartment: in the model, this process is governed by the parameter $\gamma_{\mathrm{EGFR}}$ (Figure 1 and Figure 3, Equation (10)); (2) G1 arrest antagonizes the proliferation-dependent cytotoxicity of chemotherapy [12] by blocking the X_1_→X_2_ transition (Equations (10) and (11)), keeping cells quiescent in X_1_, where they neither proliferate nor enter damaged tumor cell compartments (Figure 1 and Figure 3). This process was governed by the parameter $\gamma_{G1}$. (3) Another is the pro-apoptotic effect (Figure 1 and Figure 3): OSI downregulates Rad51 [12], thereby exacerbating PEM-induced DNA damage [56,57], and upregulates the pro-apoptotic protein Bim (the most potent pro-apoptotic protein induced by EGFR-TKIs [38,58,59,60,61]), thereby promoting PEM-induced apoptotic priming. This effect is mediated by the inhibition of MAPK signaling [13]. We grouped these mechanisms as OSI-promoted apoptosis of damaged tumor cells, which is represented by accelerating tumor-cell transit through the X_2_, X_3_, and X_4_ compartments and enhancing elimination from X_4_ (Figure 1 and Figure 3). The corresponding differential equations are given by Equations (11)–(13), and they are governed by PD parameters such as $k_{bim}$ and $\gamma_{\mathrm{bim}}$. (4) In addition to reducing EGFR signaling, OSI can directly suppress PEM-induced rebound in EGFR signaling (Figure 1 and Figure 3, Equation (9)), thereby blocking the associated pro-survival response in NSCLC cells.

The real-time in vivo tumor growth inhibition (TGI%) profile for this model was also established by comparing the drug-treated group with the unperturbed group, as described in Supplementary Method S1.5.

#### 2.3.5. Model Characterization of the Sequence-Dependent Synergistic Effects

The PEM→OSI strategy achieves synergy through a sequential pharmacodynamic priming and amplification mechanism: PEM initiates, and OSI boosts. The PEM first drives proliferating tumor cells into a damaged state (Equations (10) and (11)), which in turn triggers compensatory feedback and rebound activation of the EGFR pathway (Equation (9)). Subsequently, upon OSI exposure, OSI promoted the apoptosis of damaged tumor cells (Equations (10)–(13)) while suppressing EGFR signaling rebound (Equation (9)). An appropriate sequential interval is essential, as PEM cytotoxicity hinges on depleting folate-metabolizing enzymes, folate, and one-carbon units and requires the first conversion of proliferating cells into a damaged state to enable OSI’s synergistic action—a process that requires time. Premature OSI exposure may reduce the number of damaged tumor cells through G1 arrest, thereby compromising the subsequent synergy (Equations (10) and (11)).

In the PEM + OSI strategy, PEM’s cytotoxic process is antagonized by OSI-induced G1 arrest; therefore, cells cannot be adequately driven into a damaged state (Figure 1 and Figure 3, Equations (10) and (11)). As a result, fewer damaged tumor cells are available for OSI to promote apoptosis, and the subsequent synergy between OSI and PEM is therefore limited.

#### 2.3.6. Estimation of Important PD Parameters from In Vitro and In Vivo PD Data

The key PD parameters in the model were estimated by developing a simplified mechanistic PD sub-model (mini-model, as shown in Figure 7) and fitting it to in vitro pharmacology data. Parameter estimation was performed using a stepwise calibration strategy [62,63,64].

To determine the EGFR receptor turnover rate ($k_{\mathrm{out},\mathrm{EGFR}}$), as shown in Figure 7E,F, HCC827 cells were treated with 50 nM OSI for 72 h to achieve complete dephosphorylation and inactivation of EGFR; re-phosphorylation occurs only after synthesis of new EGFR receptors [53]. The cells were then washed three times with PBS and cultured in RPMI 1640 supplemented with 20% FBS. At 0, 12, 24, 36, 48, 60, and 72 h after OSI removal, pEGFR and total EGFR levels were quantified by Western blotting (WB) to characterize the recovery of EGFR signaling. The recovery kinetics are described by the differential Equation (18), and model fitting of the data provided an estimate of $k_{\mathrm{out},\mathrm{EGFR}}$.

(18) $$\frac{d\left(EGFR\right)}{dt}=k_{\mathrm{out},\mathrm{EGFR}}-EGFR\cdot k_{\mathrm{out},\mathrm{EGFR}}\;\;\;\;\;\;\;\;\;\;\;\;\;\;\;\;\;\;\;\;\;\;\;\;\;\;EGFR_{0h}=0$$

The EC50 of the OSI for the target was determined by fitting a four-parameter logistic curve [62] to the OSI concentration–pEGFR/EGFR plots (Figure 7G,H, quantified by WB), yielding the culture-medium EC50 value $EC_{50,osi,medium}$. Differences in the free fraction arising from protein concentration differences between the plasma and culture medium were then adjusted using Equations (19)–(21) [65]. Under the assumption that unbound OSI concentrations are equal in plasma and culture medium, the plasma EC50 value *EC*_50,osi,plasma_ was inferred based on the free-fraction differences and medium EC50 value $EC_{50,osi,medium}$ (Equation (19)). *EC*_50,pem,medium_ and *EC*_50,pem,plasma_ values were obtained and calibrated using the same method (Figure S6, and Supplementary Method S1.6).

(19) $$EC_{50,\mathrm{osi},\mathrm{plasma}}=\frac{EC_{50,\mathrm{osi},\mathrm{medium}}\cdot f_{u,\mathrm{medium}}}{f_{u,\mathrm{plasma}}}$$

(20) $$f_{u,\mathrm{medium}}=\frac{1}{1+\frac{P_{\mathrm{medium}}\;\times \;\left(1-f_{u,\mathrm{plasma}}\right)}{P_{\mathrm{plasma}}\;\times \;f_{u,\mathrm{plasma}}}}$$

(21) $$P_{\mathrm{plasma}}=10\times P_{\mathrm{medium}}$$

where $f_{u}$ denotes the unbound fraction, *f*_u,plasma_ = 5.35% [49], and P represents the protein concentration. Assuming that the serum and plasma have the same protein concentration, the plasma protein concentration is thus tenfold that in the culture medium.

The in vivo tumor growth rate constants in the Simeoni model [54], λ_0_ and λ_1_, were obtained by fitting the model to the data from the control group (Figure 8E). Based on in vitro inhibition rate–time (TGI%) profiles (Figure 7A–D), we derived PD parameters that characterized tumor inhibition resulting from suppressed EGFR signaling and reduced folate levels (Figure 7A–D).

As shown in Figure 7A,B, to determine the relationship between EGFR levels and the tumor cell proliferation rate ($\gamma_{EGFR}$), HCC827 cells were treated with 30 nM OSI, and the tumor growth inhibition rate (TGI%) was monitored over time using the MTT assay. WB analysis confirmed that EGFR levels decreased to 0.0967 of the baseline following 30 nM OSI treatment (Figure 7G,H). This EGFR level was then incorporated into the differential Equations (22)–(24) to fit the PD parameter $\gamma_{EGFR}$, which characterizes EGFR-signaling-driven tumor cell proliferation.

(22) $$\frac{dX_{1}}{dt}=k_{\mathrm{ng}}X_{1}\cdot EGFR^{\gamma_{EGFR}}$$

(23) $$\frac{dX_{2}}{dt}=k_{\mathrm{ng}}X_{2}$$

(24) $$TGI\%=\left(1-X_{1}/X_{2}\right)\times 100\%$$

In vitro tumor cell proliferation was modeled using an exponential growth model (as shown in Equations (22) and (23)). Using the reported relationship λ_0,in vitro_ = 4.736 × λ_0,in vivo_ [63] and the estimated λ_0,in vivo,HCC827_ = 0.1032/day, we calculated λ_0,in vitro_ as 0.4888/day, which corresponds to the hourly growth rate of *k*_ng,in vitro_ = 0.0203/h.

As shown in Figure 7C,D, to model the cytotoxicity resulting from deviations in folate levels from baseline, HCC827 cells were treated with 300 nM PEM (0.1282 μg/mL) for varying durations, and the tumor growth inhibition rate (TGI) was quantified using the MTT assay. The TGI was measured at 24, 48, 72, 96, and 120 h after treatment. At this PEM concentration, the TGI–time profile is described by differential Equations (25)–(32), with the PEM concentration substituted into the model to estimate folate-associated cytotoxicity parameters ($E_{\max,\mathrm{pem}}$, $\gamma_{enzyme}$, and $E_{\max,\mathrm{folate}}$).

(25) $$\frac{dX_{1}}{dt}=k_{\mathrm{ng}}X_{1}-k_{1}X_{1}\left(1+\frac{E_{\max,\mathrm{folate}}\left(1-folate\right)}{EC_{50,\mathrm{folate}}+\left(1-folate\right)}\right)$$

(26) $$\frac{dX_{2}}{dt}=k_{1}X_{1}\left(1+\frac{E_{\max,\mathrm{folate}}\left(1-folate\right)}{EC_{50,\mathrm{folate}}+\left(1-folate\right)}\right)-k_{2}X_{2}$$

(27) $$\frac{dX_{3}}{dt}=k_{2}X_{2}-k_{2}X_{3}$$

(28) $$\frac{dX_{4}}{dt}=k_{2}X_{3}-k_{2}X_{4}$$

(29) $$\frac{dX_{5}}{dt}=k_{\mathrm{ng}}X_{5}-k_{1}X_{5}$$

(30) $$TGI\%=\left(1-\left(X_{1}+X_{2}+X_{3}+X_{4}\right)/X_{5}\right)\times 100\%$$

(31) $$\frac{dEnzyme}{dt}=k_{\mathrm{out},\mathrm{enzyme}}-k_{\mathrm{out},\mathrm{enzyme}}\cdot Enzyme\cdot (1+\frac{E_{\max,\mathrm{pem}}C_{\mathrm{pem}}}{EC_{50,\mathrm{pem}}+C_{\mathrm{pem}}})$$

(32) $$\frac{dFolate}{dt}=Enzyme^{\gamma_{Enzyme}}k_{\mathrm{out},\mathrm{folate}}-k_{\mathrm{out},\mathrm{folate}}\cdot Folate$$

We analyzed the levels of Bim, 1–EGFR (i.e., EGFR deviation from baseline), and the apoptotic signal of Cleaved Poly ADP-ribose Polymerase (CL-PARP, a representative marker for cellular apoptosis signal) induced by 48 h of OSI treatment at different concentrations (Figure 7G–L), and we found a strong linear relationship between 1−EGFR and Bim (Figure 7J). Therefore, to simplify the model structure, 1−EGFR was used as a surrogate for BIM. Both Bim and 1−EGFR showed power-law relationships with their corresponding CL-PARP levels (Figure 7L). By fitting 1−EGFR versus CL-PARP, we estimated the parameter $\gamma_{bim}$, thereby linking EGFR signaling to the apoptosis signal triggered by the pro-apoptotic effect of OSI.

Other model parameters were derived from the literature [28,29,53,54], data fitting, and the assumed values. The complete and detailed differential equations, algebraic relationships for the special compartment, model parameter values, initial values in each compartment, and reaction schemes are provided in Tables S17–S21, respectively.

#### 2.3.7. Model Simulation and Global Sensitivity Analysis

All model simulations were performed using the MATLAB R2024a SimBiology toolbox (MathWorks, Natick, MA, USA) with an ODE15s Solver [28,29]. The model structure in the form of SimBiology is illustrated in Figure S5. The pattern search or lsqnonlin optimization method was used for parameter estimation. We performed a global sensitivity analysis using Sobol indices [66], with the tumor growth inhibition rate (TGI%) on day 18 as the output metric. Sobol indices were calculated based on 1000 samples generated in SimBiology within 0.5–2 times the baseline values of each parameter (2/3–3/2 times for $E_{\max,\mathrm{folate}}$ and $k_{\mathrm{out},\mathrm{enzyme}}$) in order to identify key sensitive parameters. The first-order Sobol index was used to assess the impact of a single parameter change on the final indicator, whereas the total-order Sobol index was used to evaluate the interaction of a parameter with other parameters on the final indicator.

### 2.4. Statistical and Non-Compartmental Analysis

All data are presented as the mean ± SD. Statistical analyses and plotting were performed using GraphPad Prism 8.0 (GraphPad Software, San Diego, CA, USA) and R (v4.5.1; R Foundation for Statistical Computing, Vienna, Austria) with RStudio 2025.09.2. Differences between two groups were analyzed using unpaired Student’s *t*-test. Dose–response curves were fitted using Prism with a four-parameter variable-slope logistic model. The number of biological replicates (*n*) and significance thresholds are provided in the legends of each Figure. Non-compartmental analyses (NCAs) were conducted using Phoenix WinNonlin 8.3.5 (Certara, Princeton, NJ, USA).

## 3. Results

### 3.1. No Experimentally Detectable PK-DDI Between PEM and OSI in Systemic Plasma Concentrations, Cellular Uptake, and Tumor Tissue Distribution

The cellular uptake of PEM and OSI under monotherapy and combination treatment is shown in Figure 4. At 100 μM PEM and 1 μM OSI, neither simultaneous nor sequential dosing (8 h pre-exposure to OSI) altered PEM uptake across the four NSCLC cell lines (Figure 4A), and PEM likewise did not affect OSI uptake (Figure 4B). PEM uptake showed substantial variability, likely due to its high aqueous solubility, transporter-dependent entry, and low intracellular accumulation. Trace environmental contamination may also contribute this phenomenon, indicating the need for more stringent washing and more sensitive LC-MS/MS quantification in cell lysates.

Systemic plasma concentration–time profiles of PEM and OSI administered alone or in combination in rats and two tumor-bearing mouse models (PC9-Balb/c nude and NCI-H1975-NOD-SCID) are shown in Figure 5A,B,D,E. Geometric mean ratios of key PK parameters were within 0.8–1.25 across groups, except that rats receiving PEM + OSI showed a 1.31-fold higher OSI AUC than OSI alone, but this was not statistically significant (Figure 5B). In mice, the concentration–time profiles were similar, with no significant differences in *C*_max_ or *AUC* (Figure 5D,E). These results suggest no meaningful systemic PK interaction between PEM and OSI, consistent with prior reports that no PK-DDI was observed when PEM was combined with almonertinib [3]. Given the clinical use of daily OSI, we further evaluated whether the 7-day OSI pretreatment affected PEM PK in rats and observed no change in the PEM PK profile (Figure 5C), supporting the absence of plasma-level DDI after short or prolonged co-administration.

To examine whether OSI increases intratumoral PEM exposure via vascular normalization, as reported for the structurally highly related aumolertinib [3] (Figure 5F), we assessed intratumoral PEM distribution after 7-day OSI dosing in NCI-H1975 NOD-SCID mice (tumor volume ~800 mm^3^ at the beginning of the experiments). Unlike aumolertinib, which significantly increased intratumoral exposure to PEM [3], OSI pretreatment was experimentally observed to reduce PEM concentrations in both plasma and tumor tissue. However, tumor-to-plasma ratios (*K*_tp_) were comparable between the two groups (Figure 5G), indicating no marked change in the relative tumor distribution of PEM. Because tumor volumes differed substantially between the OSI-pretreated and control groups at the beginning of the PEM tumor-tissue distribution experiment, the cause of the lower absolute PEM exposure in OSI pre-treated group cannot be conclusively determined from the present data. One possible explanation is a difference in apparent distribution (*V*) associated with tumor burden. Assuming that clearance (*CL*) remains unchanged, a smaller *V* in the OSI group would yield a larger elimination rate constant (k=CL/V), which in turn could account for the faster decline of PEM in the plasma and tumor, but $k_{\mathrm{tp}}$ was unaffected. Conservatively, if prolonged OSI reduced intratumoral PEM exposure, a sequential PEM→OSI regimen may be preferable to avoid reduced PEM exposure resulting from OSI pretreatment or concurrent exposure.

### 3.2. In Vivo Anti-Cancer-Efficacy and Safety Evaluation of PEM-OSI Combination Under Different Combination Strategies

**Figure 6:**
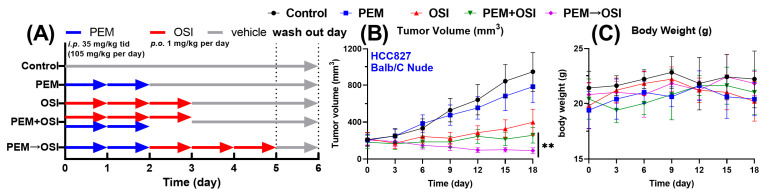
**In vivo anti-cancer efficacy and safety evaluation of PEM-OSI combination under PEM→OSI sequential and PEM + OSI concurrent strategies.** (**A**) Schematic illustration of the dosing regimen for each group in one treatment cycle; a total of three cycles were employed. (**B**,**C**) Tumor volume (**B**) and body weight changes (**C**) of HCC827 xenograft mice treated with PEM and OSI alone or in combination with different combination strategies (*n* = 5). The data in this figure is replotted from our previous reports [12]. ** *p* < 0.01.

The experimentally observed in vivo antitumor efficacy results are summarized in Figure 6. The concurrent PEM + OSI regimen produced a moderate synergistic effect, whereas the sequential PEM→OSI regimen elicited a strong synergy (Figure 6B). At the study endpoint, the mean tumor volumes in the PEM + OSI and PEM→OSI groups were reduced to 64.5% and 22.3%, respectively, of those in the OSI monotherapy group. The difference in tumor volume between the PEM + OSI and PEM→OSI regimens was statistically significant (Figure 6B). Body weight remained stable in all groups throughout the 3-week treatment period (Figure 6C), indicating no apparent toxicity for either concurrent PEM + OSI or sequential PEM→OSI regimens. This favorable safety profile was further supported by H&E-stained liver and kidney sections that showed no detectable pathological abnormalities (Figure S3).

### 3.3. Model Development and Performance of the Mechanistic QSP–PK–PD Model for Sequence-Dependent PEM–OSI Synergy Effects

Although a simple two-compartment model was used, the PK models for PEM and OSI adequately captured the PK behavior of the two drugs in PC9-bearing-Balb/c Nude mice (Figure S4A,B). Based on this, we simulated the concentration–time profiles of PEM and OSI in groups receiving monotherapy or different combination strategies (Figure S4C–F).

The tumor growth parameters in the Simeoni model were estimated by fitting the control group data (Figure 8E). The exponential phase rate constant $\lambda_{0}$ was 0.1032/day, which is very close to the literature-reported value of 0.100/day [67]. The linear-phase proliferation rate constant $\lambda_{1}$ was 51.08 mm^3^/day, which falls within a two-fold range of the reported value (35.1 mm^3^/day) [67]. These results indicated that the HCC827 tumor-bearing mice used in this study were in a normal physiological state and were suitable for in vivo inhibition efficacy evaluation and PK/PD modeling.

**Figure 7:**
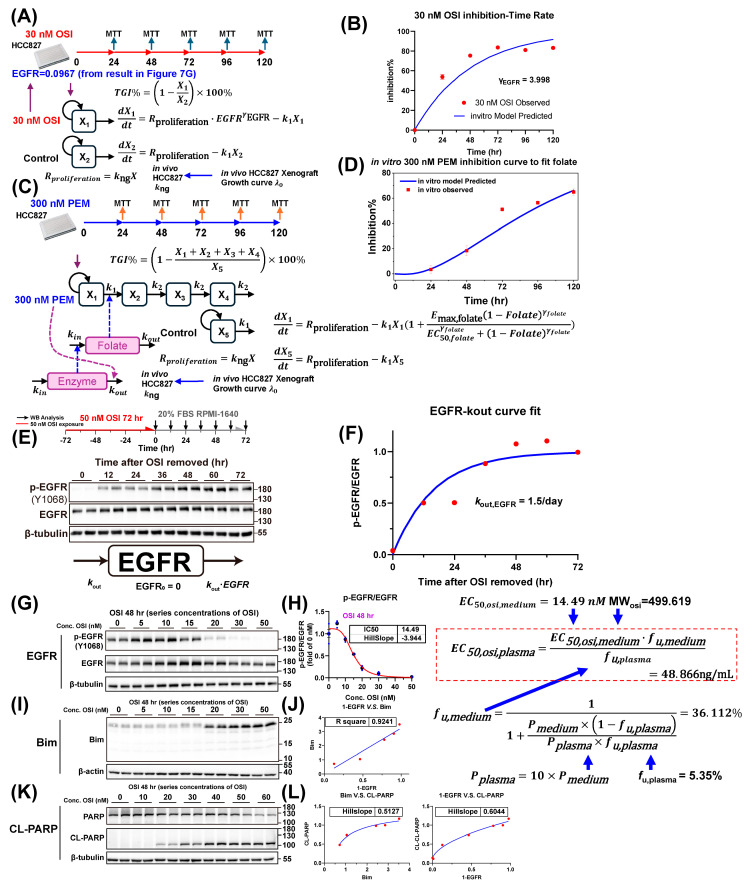
**Estimation of key pharmacodynamic (PD) parameters from in vitro PD data.** (**A**,**B**): Estimation of $\gamma_{\mathrm{EGFR}}$ based on tumor growth inhibition (TGI)–time course curve for HCC827 cells treated with 30 nM OSI in vitro. (**C**,**D**): Estimation of PD parameters for folate-related cytotoxicity from the TGI–time course curve for HCC827 cells treated with 300 nM PEM in vitro. (**E**,**F**): Estimation of the EGFR turnover rate in HCC827 cells. The cells were first treated with 50 nM OSI for 72 h to deplete EGFR signaling, washed three times with PBS, and then cultured in drug-free medium supplemented with 20% FBS. EGFR signal recovery was monitored by Western blot (WB), and the turnover rate was derived by fitting the recovery curve. (**G**,**H**): In vitro OSI concentration–EGFR inhibition curve used to estimate the EC50,osi,medium. The plasma-relevant EC50,osi,plasma was subsequently extrapolated, adjusting for differences in protein binding between culture medium and plasma and incorporating the free fraction of OSI in plasma and the protein concentrations in both matrices. (**G**–**L**): Analysis of the relationship between OSI concentration, EGFR inhibition level, Bim protein level, and apoptosis signal intensity (indicated by cleaved PARP and CL-PARP). The WB image was reproduced from our previous work [12], while modeling was performed in the present study. All data are expressed as mean ± SD.

As reported by Chen et al. in their study of deoxypodophyllotoxin, although discrepancies exist between in vitro and in vivo tumor proliferation parameters, in vitro-derived PD parameters can be reliably used to predict in vivo tumor inhibition via a PK/PD modeling approach [63]. In addition, Zhu et al. found that PD parameters obtained from patient-derived tumor organoid (PDTO) systems could effectively predict the clinical response to Oxaliplatin and Irinotecan [62]. Based on this theory, we applied an in vitro–in vivo extrapolation (IVIVE) approach to translate the PD parameters from in vitro experiments to in vivo conditions in tumor-bearing mice. The estimated EGFR receptor turnover rate in the HCC827 cell line was 1.5 day^−1^ (Figure 7E,F), which closely matches the value reported for in vivo PK/PD modeling of OSI in A431 tumor-bearing mice ($k_{rec,A431}$ = 1.44 day^−1^ [53]) and in SPC-A-1 tumor-bearing mice ($k_{\mathrm{out},\mathrm{EGFR}}\;$=1.3 day^−1^ [68]). Notably, A431 and HCC827 are both EGFR high-expressing cell lines [69]. This close agreement between the in vitro-derived $k_{out,EGFR}$ and previously reported in vivo estimates supports the feasibility of estimating tumor EGFR turnover rates from in vitro signaling recovery experiments.

**Figure 8:**
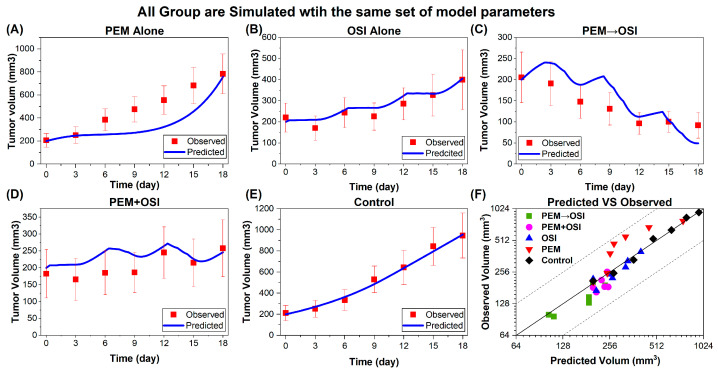
**Model-predicted tumor volume and observed tumor volume in control, PEM, OSI, PEM→OSI, and PEM + OSI groups.** (**A**–**E**): Model-predicted (blue lines) vs. observed (red symbols) tumor volumes in PEM alone (**A**), OSI alone (**B**), sequential PEM → OSI (**C**), simultaneous PEM + OSI group (**D**), and control group (**E**). (**F**): Observed vs. predicted tumor volumes; solid and dashed lines indicate the line of unity and the 2-fold prediction error margins, respectively. All data are presented as mean ± SD.

Logistic fitting of the OSI concentration–pEGFR/EGFR relationship indicated an EC50 of 14.49 nM for EGFR inhibition in the culture medium (Figure 7G,H), which is consistent with published IC50 values for OSI from in vitro studies and in vivo PK/PD modeling in tumor-bearing mice (17 and 15 nM, respectively [53]), further supporting the IVIVE strategy. After adjusting the unbound fraction based on protein concentration differences, a predicted *EC*_50,OSI,plasma_ of 48.86 μg/L was obtained, which is within two-fold of the literature reported value (84.94 μg/L) [70]. *EC*_50,pem,medium_ and *EC*_50,pem,plasma_ were obtained and calibrated using a similar method (Supplementary Method S1.6 and Figure S6), yielding a predicted *EC*_50,pem,plasma_ of 0.47315 mg/L. PD parameters characterizing tumor inhibition resulting from suppressed EGFR signaling and reduced folate levels were further estimated from the in vitro tumor growth inhibition (TGI%) curve by fitting the data to a mini sub-model (Figure 7A–D), and after being incorporated into the overall model (Figure 3), these PD parameters demonstrated satisfactory predictive performance (Figure 8).

Finally, we performed a systematic analysis at the signal transduction level to elucidate the relationships between OSI concentration, EGFR levels, Bim levels, and CL-PARP levels. Western blot data showed a strong linear relationship between 1-EGFR and Bim (Figure 7G,I,J), indicating that a deviation of EGFR from the baseline proportionally upregulated the pro-apoptotic protein Bim. Bim and 1-EGFR exhibited a power-law relationship with CL-PARP, showing a relatively flat dose–response relationship at later stages (Figure 7L). This allows the EGFR signaling level to be correlated with the pro-apoptotic capacity of OSI with the PD parameter $\gamma_{\mathrm{bim}}$.

As the proposed model framework (Figure 3) is mechanistically driven and explicitly represents the underlying pharmacological processes (Figure 1) [12], it successfully reproduces sequence-dependent synergistic efficacy with the same set of parameters. Notably, sequence-dependent synergy emerged solely from the model structure and dosing schedule (Figure 3 and Figure 6A). All observations fell within a two-fold range of the corresponding model predictions (Figure 8F), indicating good predictive performance. However, future calibration and validation of the model using multiscale in vitro and in vivo data from different sources, biological systems, and levels (e.g., NSCLC cell lines, patient-derived tumor organoid systems [32,62], CDX- and PDX-bearing mice, and clinical responses) would further improve the reliability and extrapolative capability of the model predictions. Overall, this pharmacology-informed mathematical model captures the key experimental phenomena and further supports the mechanistic hypothesis proposed in our previous study [12].

### 3.4. Global Sensitivity Analysis Identified OSI Sensitivity and Bim Protein Activity as Key Determinants of the Synergistic Efficacy of the PEM→OSI Strategy

**Figure 9:**
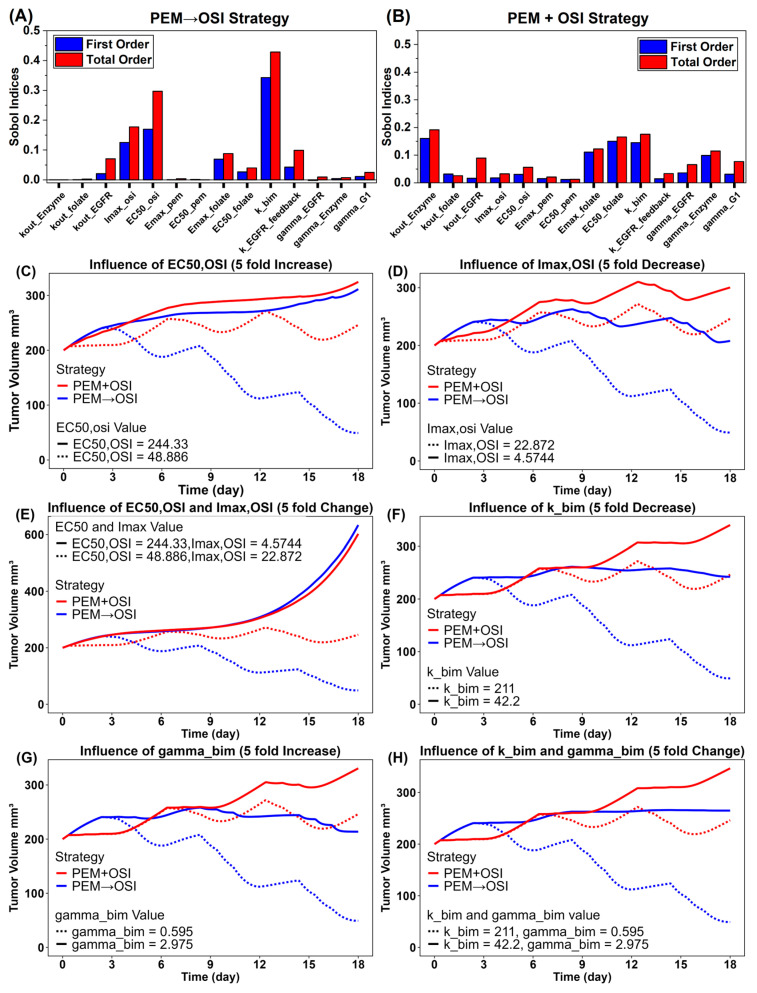
**Model-based Sobol global sensitivity analysis (GSA) and the simulated impact of OSI sensitivity and Bim protein activity on the relative advantage of PEM→OSI strategy over PEM + OSI strategy.** (**A**,**B**): Sobol global sensitivity analysis results of the PEM→OSI (**A**) and PEM + OSI (**B**) strategies. (**C**–**E**): Simulated impact of reduced OSI sensitivity on the relative advantage of the PEM→OSI strategy over the PEM + OSI strategy. (**F**–**H**): Simulated impact of reduced Bim protein activity on the relative advantage of the PEM→OSI strategy over the PEM + OSI strategy. The solid lines represent scenarios with weakened OSI sensitivity or reduced Bim protein activity, while the dashed lines represent the original scenario.

Using Sobol global sensitivity analysis [66], we identified the key parameters to which the model output was most sensitive under the sequential PEM→OSI and concurrent PEM + OSI strategies (Figure 9A,B). For PEM→OSI, the most influential parameters were $k_{\mathrm{bim}}$, $EC_{50,\mathrm{osi}}$, $I_{\max,\mathrm{osi}}$, $k_{\mathrm{out},\mathrm{EGFR}}$, $E_{\max,\mathrm{folate}}$, and $k_{\mathrm{feedback},\mathrm{EGFR}}$ (Figure 9A). For PEM + OSI, the most influential parameters were *k*_out,Enzyme_, *k*_bim_, *E*_max,folate_, *EC*_50,folate_, $\gamma_{\mathrm{Enzyme}}$, and $\gamma_{G1,\mathrm{osi}}$ (Figure 9B). Overall, the results of the sensitivity analysis show that, for PEM→OSI, NSCLC sensitivity to OSI and Bim protein activity is the primary determinant of synergistic efficacy, whereas for PEM + OSI, synergistic efficacy is jointly governed by sensitivity to both PEM and OSI (Figure 9A,B).

To validate and further explore the sensitivity analysis results, we simulated how the relative advantage of PEM→OSI over PEM + OSI varied as NSCLC cells became less sensitive to OSI. Reduced OSI sensitivity was simulated by increasing $EC_{50,\mathrm{osi}}$ fivefold, decreasing $I_{\max,\mathrm{osi}}$ fivefold, or simultaneously decreasing $I_{\max,\mathrm{osi}}$ fivefold and increasing $EC_{50,\mathrm{osi}}$ fivefold. The results (Figure 9C–E) show that as the OSI sensitivity declines, the simulated tumor inhibition curves for PEM→OSI and PEM + OSI converge, and the simulated advantage of PEM→OSI progressively diminishes and eventually disappears.

We then simulated how attenuated Bim activity alters the relative advantage of sequential PEM→OSI versus concurrent PEM + OSI. We simulated reduced Bim activity by either a fivefold decrease in $k_{\mathrm{bim}}$, a fivefold increase in $\gamma_{\mathrm{bim}}$, or both simultaneously. The simulation results are shown in Figure 9F–H. Under diminished Bim activity, the separation between the simulated tumor growth curves for PEM→OSI and PEM + OSI decreased substantially, although PEM→OSI still produced a slightly stronger simulated tumor growth inhibition (Figure 9F–H). This residual benefit likely reflects the shallow dependence of the apoptotic signal on Bim abundance (Figure 7L) such that OSI-driven Bim upregulation can partially compensate for reduced Bim activity. Overall, the simulation results suggested that attenuated Bim activity would markedly reduce the advantage of PEM→OSI over concurrent PEM + OSI.

### 3.5. Model Simulations Predicted for 48 h Are the Optimal Interval for the PEM→OSI Strategy

**Figure 10:**
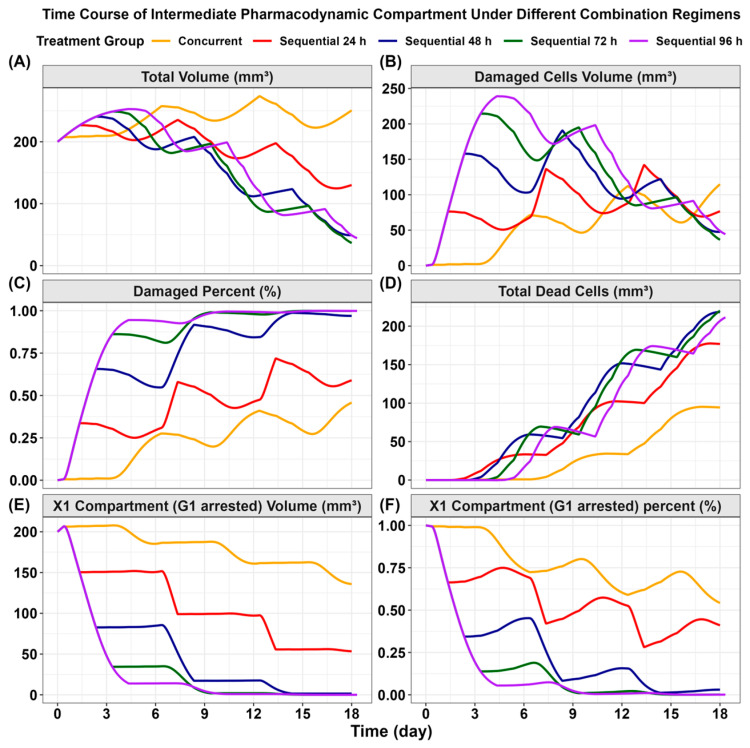
**Simulated time courses for the PEM–OSI combination under concurrent dosing or sequential PEM→OSI dosing with 24 h, 48 h, 72 h, or 96 h intervals,** showing (**A**) total tumor volume, (**B**) damaged tumor cells, (**C**) percentage of damaged tumor cells, (**D**) total dead cells, (**E**) tumor cells in the X1 compartment (indicating G1 arrested tumor cells), and (**F**) percentage of tumor cells in the X1 compartment (indicating G1 arrested tumor cells); the 48 h interval yields the optimal sequential regimen.

As illustrated in Figure 10, tumor growth curves and intermediate pharmacodynamic profiles were simulated for concurrent PEM + OSI administration and for four sequential PEM→OSI regimens with varying sequential intervals (24, 48, 72, and 96 h). The model prediction results show that all sequential dosing schedules outperformed the concurrent regimens. Notably, the 48, 72 h, and 96 h interval strategies yielded the strongest simulated tumor suppression. The final simulated tumor volume under the 48 h interval PEM→OSI schedule was less than half of that simulated with the 24 h interval, while extending the interval to 72 h or 96 h offered negligible additional benefits (Figure 10A). These simulation-based results suggest that a 48 h interval is sufficient for achieving near-maximal synergy with the PEM→OSI strategy, consistent with our earlier experimental observation that 48 h PEM exposure induces robust S-phase arrest, peaks DNA damage, and initiates apoptosis signaling beginning at 48–60 h [12].

From a mechanistic perspective, the model simulation predicts that the number of damaged cells plateaus by 48 h; thus, extending the interval further may not increase the damaged cell population, nor does it allow subsequent OSI to produce a stronger synergistic effect. Consistent with this, the 48, 72, and 96 h interval PEM→OSI regimens resulted in nearly identical proportions of simulated damaged tumor cells, and they were significantly higher than those achieved with concurrent PEM + OSI or the 24 h interval PEM→OSI (Figure 10C). The simulated total tumor cell clearance with these three regimens was nearly double that of the concurrent regimen (Figure 10D). Analyses of simulated G1-arrested cells further showed that concurrent PEM + OSI may lead to cell cycle-interfering antagonism, attributable to the high proportion of G1-phase cells (Figure 10E,F). Although a 24 h interval PEM→OSI strategy may reduce this proportion through a “pharmacodynamic separation” strategy [14,15], it did not fully avoid antagonism. In contrast, a 48 h interval markedly decreased G1-phase cells, and extending the interval to 72 or 96 h offered no additional benefit (Figure 10E,F). Importantly, these simulations suggest that an optimized sequential schedule does not require prolonged OSI interruption, thereby alleviating clinical concerns that an extended OSI drug holiday might risk disease progression.

### 3.6. Monte Carlo Simulations Comparing PEM→OSI and PEM + OSI Under BIM Deletion Polymorphism and Inter-Individual Variability in Drug Sensitivity

Based on the reported prevalence of BIM deletion polymorphism in East Asian patients (11.5%, CTONG0901 [71]), we evaluated the advantage of the PEM→OSI strategy over the PEM + OSI strategy at the population level using a Monte Carlo simulation approach [62,72,73], accounting for both BIM deletion polymorphism and inter-individual variability in tumor growth and drug sensitivity, with detailed procedures described in Supplementary Method S1.7. The simulated objective response rate (ORR)–time curve was generated based on simulations over 210 days. The simulated ORR was calculated as the number of responders divided by the total number of virtual subjects. A responder was defined as a subject whose simulated tumor volume decreased to 70% or less of its initial tumor volume according to Response Evaluation Criteria in Solid Tumors version 1.1 (RECIST 1.1) [74].

To simulate the Bim-deleted genotype, Bim activity was assumed to be reduced to 10% of that in the wild-type genotype ($k_{bim}$ = 21.1), and $\gamma_{bim}$ was assumed to increase fivefold. A total of 2000 virtual mice were generated. For $k_{bim}$ and $\gamma_{bim}$, a two-point distribution was first used to represent the proportions of the two genotypes in the population. Subsequently, 30% log-normally distributed inter-individual variability was applied to each $k_{bim}$ and $\gamma_{bim}$ value [62,72,73]. All other PD parameters and tumor growth-related parameters were also assigned 30% log-normally distributed inter-individual variability [62,72,73].

**Figure 11:**
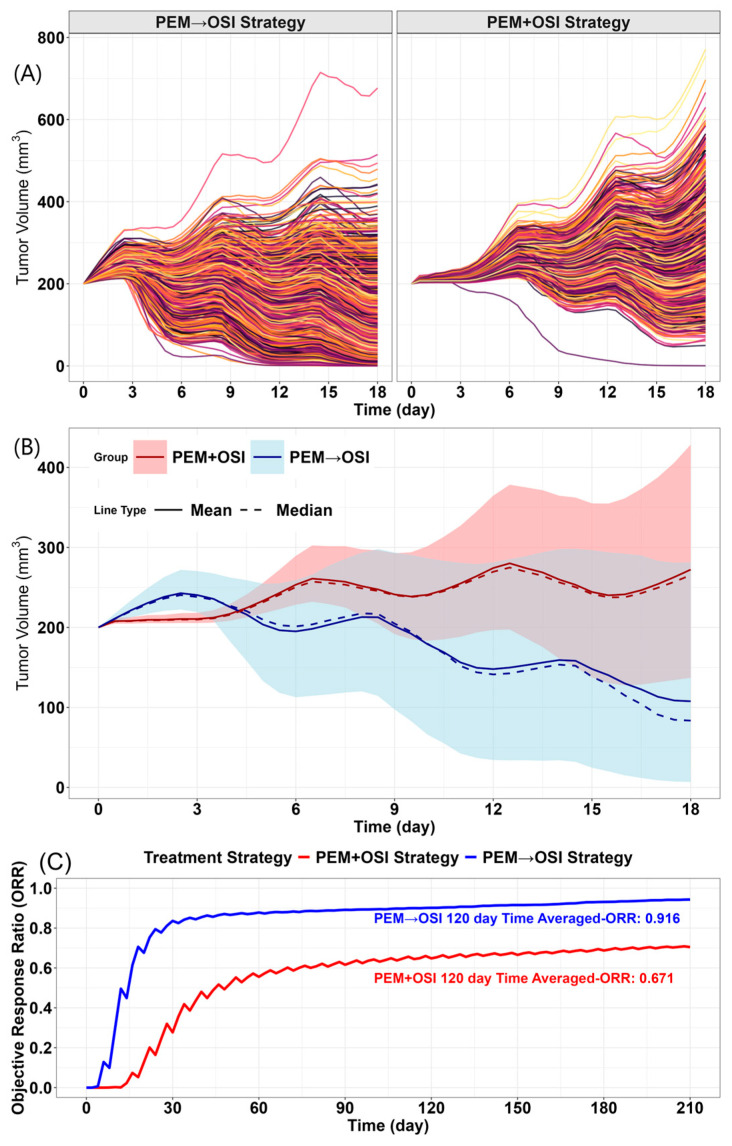
**Monte Carlo simulation results comparing the advantage of the PEM→OSI strategy over the PEM + OSI strategy,** incorporating an 11.5% prevalence of BIM deletion polymorphisms and 30% inter-individual variability in PD parameters. (**A**): Simulated tumor growth profiles of 2000 virtual mice under different combination regimens. (**B**): Simulated mean and median time–tumor volume curves with corresponding 5th–95th percentile intervals for each combination strategy. (**C**): The simulated ORR–time curve was generated based on a 210-day simulation. ORR (objective response rate) was calculated as the number of responders divided by the total number of virtual subjects. A responder was defined as a subject whose tumor volume decreased to 70% or less of its initial tumor volume according to RECIST 1.1 [74]. Individual- and population-level simulated tumor volume curves from the 210-day simulations for both strategies are shown in Figure S7.

The simulations (Figure 11) suggested that the PEM→OSI strategy remained superior to the PEM + OSI strategy at the population level despite BIM deletion polymorphism and inter-individual variability. The simulated time-averaged ORR for the PEM→OSI strategy was 91.6% (Figure 11C), which was close to the clinically reported ORR of 93.3% for sequential PEM→aumolertinib treatment [3]. Because aumolertinib is structurally highly similar to OSI, this comparison may provide an indirect clinical reference, although the regimens are not fully identical. In contrast, the simulated ORR for the concurrent PEM + OSI strategy was 67.14% (Figure 11C), which was lower than the 83% reported in FLAURA2 for OSI combined with PEM plus platinum [5] and closer to the 64% reported for aumolertinib monotherapy [3] and the 71% reported for OSI monotherapy [4]. Notably, the simulated ORR for the PEM→OSI strategy remained higher than both the simulated ORR for the concurrent PEM + OSI strategy (67.14%) and the clinically reported ORR (83%) for OSI combined with PEM plus platinum in FLAURA2 [5]. The higher ORR observed for the concurrent combination strategy in FLAURA2 may partly reflect differences in regimen composition, as platinum was included in that clinical combination strategy. These literature-based comparisons should be interpreted as indirect clinical references only, because the corresponding treatment regimens and clinical settings were not fully identical to those represented in the simulations.

## 4. Discussion

### 4.1. PK-DDI Potential Evaluation

No significant PK interactions between PEM and OSI were observed at systemic plasma exposure and tumor cellular uptake or in tissue distribution levels (Figure 4 and Figure 5). This is consistent with their distinct elimination pathways, with PEM primarily cleared by renal excretion [17,44,45,46] and OSI mainly metabolized by hepatic CYP3A4/5. Although OSI is a weak CYP3A inducer [21,35,48,49,51], the lack of overlapping clearance routes suggests a low theoretical risk of PK-DDI, while experimental confirmation remains important given the limited existing evidence.

Both drugs act on intracellular targets, and intratumoral drug exposure is more directly linked to antitumor efficacy than systemic plasma concentration [45,46,75]. Previous reports suggest that OSI may increase PEM tumor exposure through vascular normalization [3], modulation of tumor microenvironment pH toward neutrality, and inhibition of BCRP-mediated efflux [21] under acidic conditions. However, in this study, OSI did not measurably alter PEM cellular uptake or tumor distribution. This discrepancy may be partially attributed to the fact that the in vitro uptake experiments were performed at pH 7.4, and they may not adequately mimic the acidic tumor microenvironment under which the inhibition of BCRP was reported to significantly enhance PEM cellular uptake [18]. In addition, the decrease in intratumoral PEM concentrations after prolonged OSI administration, despite unchanged *K*_tp_ values, warrants further investigation (Figure 5F,G). In this study, we assumed that OSI may affects the elimination rate of PEM by altering the apparent volume of distribution ($V_{\mathrm{mice}}$); however, in the real-world clinical setting, tumor volume likely contributes only a small fraction to $V_{\mathrm{patient}}$, so OSI’s impact via this mechanism may be limited. Although both aumolertinib [3] and OSI [22] have been reported to exert potential vascular normalizing effects, the extent to which OSI modulates the tumor microenvironment remains unclear, and its impact on the intratumoral distribution of PEM warrants further study.

### 4.2. Mechanistic Interpretation of Sequence-Dependent Synergy Using the QSP-PK-PD Model

Conventional PK–PD combination models, most of which are based on the Koch framework [25], have been widely used to describe the sequence-dependent synergy between chemotherapy and targeted therapy [16,26,30]. However, these models are largely descriptive and do not explicitly incorporate drug–drug interactions into the model structure, limiting their ability to mechanistically represent synergistic processes. In these models, synergy is typically quantified by fitting the tumor growth curve to estimate a combination-strategy-specific empirical combining factor (φ), which represents the fold change in a drug’s PD parameter under a given combination strategy, with different strategies yielding different φ values. However, this approach makes it difficult to determine whether differences in the predicted inhibition among different strategies are driven by changes in the combining factor (φ) values or by the dosing schedule. As a result, such models have limited applicability for extrapolating to dosing regimens of interest (e.g., sequential strategy under different intervals) or to tumors with variant sensitivity to drugs.

In contrast, mechanistic QSP-PK-PD models are well suited for capturing complex signaling network interactions [28,29]. The QSP–PK–PD model developed in this study (Figure 3 and Figure S5) was constructed based on the pharmacological synergistic mechanisms elucidated in our previous study (Figure 1) [12]. At the structural level, the model reproduces the relay-type synergy of the PEM→OSI regimen, whereby PEM induces cytotoxic damage and drives proliferating tumor cells into damaged states (X_2_–X_4_, Figure 10C) while simultaneously upregulating EGFR signaling to promote cell survival through feedback mechanisms. OSI subsequently enhances apoptosis of damaged tumor cells and suppresses feedback-activated EGFR signaling. In addition, the model captured the antagonistic effect of OSI-induced G1 arrest on PEM cytotoxicity under concurrent PEM + OSI or 24 h interval PEM→OSI strategies (Figure 1 and Figure 10E,F). These mechanisms are mathematically illustrated in Figure 10 by comparing the simulated time-course changes in the fractions (volume %) of G1-arrested, damaged, and apoptotic tumor cells under concurrent versus sequential regimens at different intervals (24, 48, 72, and 96 h).

The complexity of QSP models necessitates a large number of PD parameters, which poses challenges for model implementation. To address this, we employed an IVIVE-based strategy to derive PD parameters (Figure 7 and Figure S6), enabling translation across in vitro and in vivo systems, as well as across biological scales. While IVIVE has been traditionally used in PBPK modeling, its application in PK/PD modeling of anti-cancer drugs has increased in recent years. Previous studies have shown that in vitro PD parameters can successfully predict tumor growth in tumor-bearing mouse models [63] and recapitulate clinical responses using patient-derived tumor organoid (PDTO) systems [62]. Inspired by these findings, we integrated IVIVE with pharmacological data from our previous studies [12] to determine PD parameters, while the remaining parameters were sourced from the literature and assumed or estimated through model fitting [28,29,53,70]. The resulting model demonstrated a satisfactory predictive performance (Figure 8), supporting the feasibility and practical utility of the proposed IVIVE-based QSP-PK-PD modeling strategy. Although some parameters lacked direct experimental or literature support and were inferred by reproducing the observed pharmacodynamic behavior, the parameters identified by sensitivity analysis as having the greatest influence on the PEM→OSI synergistic effect, including $EC_{50,\mathrm{OSI}}$, $k_{\mathrm{bim}}$, and $I_{\max,\mathrm{osi}}$, were derived from well-designed in vitro experiments or in vivo PK/PD modeling studies (Figure 7G,H and literature [53]). Therefore, the main sequence-dependent model predictions are supported by parameters with direct experimental or modeling basis, whereas uncertainty remains for some indirectly estimated parameters. Given the differences among the experimental conditions underlying the available datasets, fitted parameter values should be interpreted cautiously and may be further refined as additional calibration and validation data become available. Sensitivity analysis also suggests that some model predictions depend more strongly on selected parameter values, and thus, prospective confirmation with additional experimental or clinical data would further strengthen confidence in the model.

### 4.3. Model-Based Analysis of the Influence of OSI Sensitivity and Bim Protein Activity

Global sensitivity analysis identified OSI sensitivity and Bim protein activity as the primary determinants of the tumor inhibitory efficacy of the PEM→OSI strategy (Figure 9A), whereas the efficacy of the concurrent PEM + OSI regimen was jointly influenced by sensitivity to both PEM and OSI (Figure 9B). The simulation results showed that a five-fold reduction in OSI sensitivity progressively attenuated the superiority of the PEM→OSI strategy, eventually abolishing its advantage over PEM + OSI (Figure 9C–E). Similarly, a five-fold reduction in Bim activity substantially weakened, but did not eliminate, the simulated benefit of the sequential regimen (Figure 9F–H).

These simulation findings indicate that the PEM→OSI strategy may be the most effective strategy for OSI-naïve patients with high intrinsic OSI sensitivity. As the treatment progresses, declining OSI responsiveness is likely to compromise the efficacy of sequential combination therapy. Supporting this conclusion, OSI resistance has been reported to confer cross-resistance to PEM. La Monica et al. [9] demonstrated that OSI-resistant PC9T790M cells exhibit elevated thymidylate synthase (TS) expression, a validated sensitivity biomarker of PEM (higher TS indicates lower PEM sensitivity [76,77]), resulting in markedly reduced PEM sensitivity [9]. Mechanistically, the synergistic effect of OSI with PEM in the PEM→OSI strategy may predominantly arise from enhanced DNA damage and pro-apoptotic signaling, processes that are critically dependent on OSI sensitivity and Bim activity [12]. Consistent with previous experimental reports, EGFR-TKIs primarily induced G1 arrest in non-sensitive EGFR wild-type cells, whereas robust apoptosis was restricted to sensitive EGFR-mutant cells [14,78]. More importantly, reduced or lost BIM activity is often associated with resistance to EGFR-TKIs [79,80]. Such Bim-dependent pro-apoptotic activity is essential not only for the intrinsic antitumor efficacy of EGFR-TKIs but also for their ability to enhance PEM-induced damaged tumor cell death [79,80].

### 4.4. Simulation Insights and Clinical Implications

Model simulations identified a 48 h interval as the optimal schedule for the PEM→OSI regimen (Figure 10A). Mechanistically, PEM requires sufficient time to induce folate pathway inhibition, nucleotide depletion, S-phase arrest, replication fork stress, and progressive DNA damage [55], which collectively prime tumor cells for apoptosis. Our simulation results suggested that a 24 h interval is insufficient for these processes to fully develop (Figure 10C), whereas premature OSI administration may induce G1 arrest, thereby antagonizing PEM-driven S-phase effects (Figure 10E,F). In contrast, a 48 h interval appeared to allow the accumulation of substantial DNA damage and apoptotic priming (Figure 10C) and fully developed S-arrest while avoiding G1 arrest (Figure 10E,F), enabling subsequent OSI treatment to efficiently trigger apoptosis (Figure 10D). Extending the interval to 72 or 96 h appeared to offer no additional benefit, as PEM-induced DNA damage and apoptotic signaling plateau beyond this time window. These simulation results provide a mechanistic rationale for optimizing the PEM→OSI sequencing schedule and suggest that only a short OSI drug holiday (approximately two days) may be sufficient, thereby alleviating the clinical concern that prolonged interruptions in OSI dosing could lead to disease progression [12,13].

To better reflect clinical heterogeneity, Monte Carlo simulations of both tumor volume and ORR were performed in 2000 virtual tumor-bearing mice, incorporating variability in tumor growth rates, tumor sensitivity to PEM and OSI, and Bim-deletion polymorphisms (Figure 11 and Figure S7). Despite substantial inter-individual variability, simulations showed that the PEM→OSI strategy consistently outperformed concurrent PEM + OSI regimens at the population level, underscoring the robustness and translational potential of the sequential approach. The PFS curve [62] was not included in the simulations because modeling disease progression requires the incorporation of long-term drug resistance characteristics [81], which were not included in the current model. This represents a key direction for future refinement of the model.

### 4.5. Limitations of This Study

While our modeling and simulations results suggested that the sequential PEM→OSI strategy may provide therapeutic advantages under complex clinical scenarios and that a 48 h interval may be the optimal schedule for this regimen, several limitations of the current framework should be taken into consideration when interpreting these findings. First, as in many other modeling studies [28,29,31,32,33,34,82,83], the present framework was developed primarily on the basis of preclinical data [12] and model-based simulations rather than direct patient-level clinical observations. Therefore, the predicted therapeutic advantages of sequential regimens should be viewed as preliminary findings rather than definitive clinical evidence, and clinical confirmation is still required. In addition, external validation and calibration using fully independent multiscale in vitro/in vivo datasets remain limited. More importantly, the relative advantages of different sequential intervals have thus far been compared mainly at the cellular level [12], and stronger in vivo validation of the key model predictions, particularly the predicted superiority of the 48 h interval, would further strengthen the conclusions. The current model was established using HCC827 tumor-bearing mice, a system characterized by high EGFR expression and high sensitivity to OSI [12]. Future validation in models with different EGFR statuses and more moderate sensitivity to OSI would be important to improve the generalizability and translational relevance of the present findings. Second, two major active metabolites of OSI, AZ-5104 and AZ-7550 [53,84,85], were not explicitly incorporated into the current model. Although each accounts for less than 10% of total OSI exposure [84], incorporating their pharmacological contributions may further improve the quantitative predictive performance of the framework. Third, the current model may modestly overestimate the tumor-inhibitory effect of the PEM→OSI regimen when a 24 h interval is used, suggesting that additional optimization is still needed to better distinguish the efficacy differences between the 24 h and 48 h schedules to more precisely characterize the impact of sequential interval timing on synergistic efficacy. Finally, the present study mainly focused on short-term tumor volume dynamics and growth inhibition. Since an important rationale for combining EGFR-TKIs with chemotherapy is to delay or overcome therapeutic resistance, future extensions of the model should incorporate resistant tumor subpopulations and tumor competition dynamics [81] to enable a more comprehensive evaluation of long-term therapeutic benefit. Integrating these mechanisms may also allow predictions of the PFS curve [62] and provide a more clinically meaningful assessment of the relative advantage of the PEM→OSI strategy.

## 5. Conclusions

The combination of PEM and OSI showed no significant PK–DDI in cellular uptake, systemic plasma exposure, or tumor tissue distribution, indicating that the synergy of the PEM→OSI sequential strategy is primarily driven by pharmacodynamic interactions. The strong in vivo anticancer synergy of the PEM→OSI strategy, together with the absence of appreciable hepatorenal toxicity and lack of PK–DDI, supports its translational potential. Based on in vitro and in vivo data, supplemented with the literature’s data, we developed a QSP–PK–PD model with a unified structure and the same set of parameters that successfully reproduced the synergistic efficacy between different combination regimens, demonstrating that sequence-dependent synergy is intrinsic to the model’s structure. Model simulations identified a 48 h sequential interval as optimal, which alleviates concerns that prolonged interruption of OSI therapy could lead to disease progression. Sensitivity analysis indicated that OSI sensitivity and Bim activity may be the key determinants of the benefit of the PEM→OSI strategy. Model simulation predicted that, as OSI sensitivity decreases or Bim activity weakens, the advantage of PEM→OSI over concurrent PEM + OSI diminishes, suggesting greater suitability for EGFR-TKI-naïve patients. Notably, Monte Carlo simulations suggested that even after incorporating Bim deletion polymorphisms and inter-individual variability in drug sensitivity, PEM→OSI remained superior to PEM + OSI.

## Data Availability

The original contributions presented in this study are included in the article/Supplementary Material. Further inquiries can be directed to the corresponding author.

## Supplementary Materials

The following supporting information can be downloaded at https://www.mdpi.com/article/10.3390/pharmaceutics18040408/s1.

## Abbreviations

PEM	Pemetrexed
OSI	Osimertinib
PEM→OSI	PEM Treatment Followed by OSI Treatment
PEM + OSI	PEM and OSI Concurrent Treatment
PK	Pharmacokinetics
PD	Pharmacodynamics
LC-MS/MS	Liquid Chromatography–Tandem Mass Spectrometry
QSP	Quantitative System Pharmacology
DDI	Drug–Drug Interaction
NSCLC	Non-Small Cell Lung Cancer
EGFR	Epidermal Growth Factor Receptor
TS	Thymidylate Synthase
PFS	Progression Free Survival
OS	Overall Survival
ORR	Objective Response Rate
IC50 and EC50	Half-Maximal Efficient Concentration
NCA	Non Compartmental Analysis
TGI	Tumor Growth Inhibition
IVIVE	In Vitro–In Vivo Extrapolation
GSA	Global Sensitivity Analysis
RECIST 1.1	Response Evaluation Criteria in Solid Tumors Version 1.1
BCRP	Breast Cancer Resistance Protein, an Efflux Transporter of PEM
