# Supplementary material for: A Mechanistic Pharmacokinetic/Pharmacodynamic Model for Sequence-Dependent Synergy in Pemetrexed–Osimertinib Combinations Against Non-Small Cell Lung Cancer (NSCLC): Translational Insights
Source: Pharmaceutics. 2026 Mar 26;18(4):408. doi: 10.3390/pharmaceutics18040408 (PMC13118741; doi:10.3390/pharmaceutics18040408)
Supplement: Supplementary file 1 [file pharmaceutics-18-00408-s001.zip › pharmaceutics-4191467-Supplementary Materials.pdf]

pharmaceutics

Supplementary Materials for: A mechanistic Pharmacokinetic/Pharmacodynamic model for Sequence-Dependent Synergy in Pemetrexed–Osimertinib Combinations Against Non-Small Cell Lung Cancer (NSCLC): Translational Insights

Kuan Hu, Yan Lin, Huachun Ji, Tong Yuan, Yu Xia, Jin Yang

1. Supplementary Method

1.1 Cell line cultures and tumor-bearing mice inoculation

Four human non-small cell lung cancer (NSCLC) cell lines harboring EGFR mutations (HCC827, PC-9, NCI-H1975, and NCI-H1650) were obtained from the Cell Bank of the Chinese Academy of Sciences (Shanghai, China). All cell lines were cultured in RPMI-1640 medium (KeyGen BioTech, Nanjing, China) supplemented with 10% fetal bovine serum (FBS; Procell, Wuhan, China), 1% GlutaMAX, and penicillin–streptomycin (100 U/mL penicillin and 100 μg/mL streptomycin). Cells were maintained at 37°C in a humidified incubator with 5% CO_2. Cell line authentication was confirmed by short tandem repeat (STR) profiling performed by an accredited commercial provider (Genetic Testing Biotechnology Corporation, China), and the STR report is presented in Figure S8, S9, S10.

In this study, PC9-bearing BALB/c nude mice and NCI-H1975–bearing NOD/SCID mice were used for PK–DDI studies, whereas HCC827-bearing BALB/c nude mice were used to evaluate *in vivo* anticancer efficacy. Tumor cells were washed once with PBS, resuspended in serum-free medium, and mixed 1:1 (*v/v*) with high-concentration Matrigel. The resulting suspension (0.2 mL per mouse) was inoculated subcutaneously into the right flank of the mice. The numbers of cells inoculated per mouse were 5×10^6 for PC9, 5×10^6 for NCI-H1975, and 1×10^7 for HCC827.

1.2 Quantitative PEM in bio-matrix by LC-MS/MS

The PEM and OSI concentrations in cell lysates, tumor tissue homogenates, and plasma were analyzed using LC-MS/MS. The LC-MS/MS system consisted of a Shimadzu LC-20AD HPLC system and an API-4000 triple quadrupole mass spectrometer equipped with an ESI ion source operated in selective reaction monitoring (SRM) transition mode.

For the analysis of PEM, 1 mL of precipitants (methanol containing 0.2 μg/mL imatinib as internal standard) was used to precipitate 10 μL of plasma and tumor tissue homogenate ; 200 μL of precipitant was used to precipitate 50 μL of cell lysates. After protein precipitation, samples were vortexed for 5 min and centrifuged at 13,000 × *g* for 5 min. 2 and 20 μL supernatants were injected for the analysis of plasma/tumor tissue homogenates and cell lysate samples, respectively.

Chromatographic separation was performed on an ACE C18 Amide column (2.0 mm × 100 mm, 3 μm). Mobile phase A was water containing 0.1% FA and mobile phase B was methanol. The flow rate was 0.3 mL/min, and the column temperature was set at 40°C. The gradient program was as follows: 0–0.1 min, 5% B; 0.1–4.3 min, 5–55% B; 4.3–4.5 min, 55–95% B; 4.5–5.5 min, 95% B; 5.5–5.7 min, 95–5% B; 5.7–7.5 min, 5% B. (Table S5, S6)

The Mass spectrometry parameters for PEM are listed in Table S3 and S4. Typical SRM chromatograms of PEM are shown in Figure S1.

The Calibration point setting for PEM in different bio-matrix were listed in Table S7.

1.3 Quantitative OSI in bio-matrix by LC-MS/MS

For the analysis of OSI in plasma, tumor tissue homogenate, and cell lysate, 0.5 mL of precipitant (methanol containing 0.02 μg/mL imatinib and 0.002 μg/mL erlotinib as internal standard) was used to precipitate 10 μL of biological matrix. After protein precipitation, the samples were vortexed for 5 min, centrifuged at 13,000 × *g* for 5 min, and 3 μL of supernatant was injected for analysis.

Chromatographic separation was performed on an ACE Super C18 column (2.0 mm × 100 mm, 3 μm). Mobile phase A was water containing 0.1% FA and 10 mM ammonium acetate, and mobile phase B was acetonitrile containing 0.1% FA. The flow rate was 0.5 mL/min and the column temperature was set at 40°C. The gradient program was as follows: 0–0.1 min, 5% B; 0.1–3.5 min, 5–75% B; 3.5–3.7 min, 75–95% B; 3.7–5.8 min, 95% B; 5.8–6.0 min, 95–5% B; 6.0–7.0 min, 5% B (Table S10 and S11).

The Mass spectrometry parameters for OSI are listed in Table S8 and S9. Typical SRM chromatograms of OSI are shown in Figure S2.

The Calibration point setting for OSI in different bio-matrix were listed in Table S12.

1.4 Consideration for concentrations and doses selection in PK-DDI study

The doses or concentrations of PEM and OSI used in the different PK-DDI experiments were selected according to the objective of each study; therefore, the PEM/OSI ratios were not intended to be identical across experiments.

In the *in vivo* PK-DDI studies in tumor-bearing mice and rats, our main objective was to assess the potential for PK interaction under literature-supported and pharmacologically relevant dosing conditions. In the tumor-bearing mouse PK-DDI study, PEM and OSI were given at 100 mg/kg and 5 mg/kg, respectively, based on published reports [1-5]. We then conducted a pilot study in rats and found that PEM 70 mg/kg plus OSI 10 mg/kg produced a PEM/OSI exposure relationship similar to that observed in mice, with an AUC ratio of approximately 1:8–1:10 (noting that PEM concentrations were expressed in μg/mL and OSI concentrations in ng/mL). Therefore, PEM 70 mg/kg and OSI 10 mg/kg were selected for the rat PK-DDI study to achieve a systemic exposure relationship comparable to that in mice.

In the cellular uptake PK-DDI study, our aim was to evaluate whether PEM and OSI might interact at the level of tumor cell uptake under clinically relevant high-exposure conditions. Therefore, the test concentrations were selected mainly with reference to the highest plasma concentrations achievable in humans and animals. Our rationale was that if OSI did not alter PEM uptake under near-maximal exposure conditions, the likelihood of an uptake-level PK-DDI would be low. According to population PK studies [6,7], the $C_{\max}$ of OSI in the overall patient population is approximately 1 μM [7] (1000 nM, about 500 ng/mL), whereas the $C_{\max}$ of PEM is close to 100 μM [6] (about 42741.7 μg/L). In addition, LC-MS/MS sensitivity was an important practical consideration. Because PEM has low lipophilicity and its cellular uptake is highly transporter-dependent (e.g., RFC, PCFT, and BCRP), a relatively high PEM concentration was required for reliable detection in cell lysates. Based on both clinically relevant exposure levels and analytical feasibility, we selected 100 μM PEM and 1 μM OSI for the cellular uptake PK-DDI study.

1.5 in vivo Real-time TGI% (tumor growth inhibition%) profile generating

To describe the *in vivo* real-time TGI% (tumor growth inhibition%) profile, we also included an unperturbed group in the model in which tumor growth was not affected by drug intervention. Except for the absence of drug effects, the meanings of all compartments and the parameter values are identical to those in the regular group. This unperturbed group is described by the differential Equation. (S1-6) as follows:

$$\frac{dX_{1,\text{unperturbed}}}{dt} = \frac{\lambda_0 X_{1,\text{unperturbed}}}{\left[1 + \left(\frac{\lambda_0}{\lambda_1} X_1\right)^{\psi}\right]^{\frac{1}{\psi}}} - k_1 X_{1,\text{unperturbed}} \tag{S1}$$

$$\frac{dX_{2,\text{unperturbed}}}{dt} = k_1 X_{1,unperturbed} - k_2 X_{2,\text{unperturbed}} \tag{S2}$$

$$\frac{dX_{3,\text{unperturbed}}}{dt} = k_2 X_{2,\text{unperturbed}} - k_2 X_{3,\text{unperturbed}} \tag{S3}$$

$$\frac{dX_{4,\text{unperturbed}}}{dt} = k_2 X_{3,\text{unperturbed}} - k_2 X_{4,\text{unperturbed}} \tag{S4}$$

$$X_{\text{unperturbed}} = X_{1,\text{unperturbed}} + X_{2,\text{unperturbed}} + X_{3,\text{unperturbed}} + X_{4,\text{unperturbed}} \tag{S5}$$

$$TGI\% = 1 - \left(X/X_{\text{unperturbed}}\right) \tag{S6}$$

1.6 Estimation of in vivo EC$_{50,pem,plasma}$

We first evaluated $EC_{50,\text{pem,medium}}$ (Figure S6). In clinical practice, the plasma concentration of PEM remains above the effective level for no more than 24 h, indicating that tumor cells *in vivo* are exposed to relatively high PEM concentrations for at most 24 h. Accordingly, $EC_{50,\text{pem,medium}}$ was determined under a scenario in which cells were exposed to different concentrations of PEM for 24 h, washed three times with 1× PBS, and then cultured in drug-free medium for an additional 72 h (to evaluate late-phase inhibitory effects resulting from a short-term PEM exposure, as quantified by the EC_{50}). The estimated $EC_{50,\text{pem,medium}}$ was 300 nM (MW_{pem} = 427.417, 300 nM PEM = 0.1282251 mg/L).

Assuming that the free PEM concentrations in plasma and culture medium are identical, differences in free fraction were first corrected based on differences in protein concentration (Equation. S8 and S9). The corrected free fraction was then used to extrapolate $EC_{50,\text{PEM,plasma}}$ from $EC_{50,\text{PEM,medium}}$ (Equation. S7):

$$EC_{50,\text{pem,plasma}} = \frac{EC_{50,\text{pem}} \cdot f_{\text{u,medium}}}{f_{\text{u,plasma}}} \tag{S7}$$

$$f_{\text{u,medium}} = \frac{1}{1 + \frac{P_{\text{medium}} \times \left(1 - f_{\text{u,plasma}}\right)}{P_{\text{plasma}} \times f_{\text{u,plasma}}}} \tag{S8}$$

$$P_{\text{plasma}} = 10 \times P_{\text{medium}} \tag{S9}$$

$f_{\text{u,pem,plasma}}$=0.19 (according to the drug instructions for PEM)

Using this approach, the final estimated $EC_{50,\text{pem,plamsa}}$ was 0.47315 mg/L.

1.7 Generating Virtual Tumor Bearing Mice and Monte Carlo Simulation

During the Monte Carlo simulation, we first generated 2,000 virtual tumor-bearing mice with distinct PD parameter sets. Batch simulations were then performed using SimBiology's "Generate Samples → Simulation" workflow by importing these 2,000 virtual subjects via "User-Specified Values from a data table". The simulation results were exported for statistical analysis using the tidyverse package in R, and graphical visualization was performed using the ggplot2 package.

For PD parameters of the virtual tumor-bearing mice that were not related to Bim activity, individual parameter values were generated by introducing inter-individual variability around the typical values. We assumed that these PD parameters follow a log-normal distribution. Accordingly, an individual parameter value P can be expressed as shown in Equation. S10, where η follows a normal distribution with a mean of 0 and a variance of σ (Equation. S11), and σ was calculated using Equation. S12.

$$P = P_{\text{TV}} \cdot e^{\eta} \text{a} = 1, \tag{S10}$$

$$\eta \sim N(0, \sigma) \tag{S11}$$

$$\sigma = \sqrt{ln(1 + CV^2)} \tag{S12}$$

Random PD values were generated using the formula function in Microsoft Excel, spanning a total of 2,000 cells: "=PTV*EXP(NORM.INV(RAND(),0,SQRT(LN(1+CV^2))))"

Here, PTV denotes the typical value of the parameter to be simulated, and CV represents the coefficient of variation of the target distribution.

For Bim activity–related PD parameters (k_{bim} and $gamma_{\mathrm{bim}}$) in the virtual tumor-bearing mice, a similar procedure was applied, with an additional step to account for Bim deletion mutations. Specifically, the Bim deletion status of each virtual mouse was first randomly assigned using the formula in the State cell:

"=IF(RAND()<0.885,0,1)".

In this setting, 0.885 represents the frequency of non–Bim deletion mutation (88.5%). An output of 0 indicates absence of the deletion mutation, whereas an output of 1 indicates presence of the deletion mutation.

Subsequently, the Bim-related parameter value for each virtual mouse was randomly generated using the following formula:

"=IF(State=0,P_normal,P_delete)*EXP(NORM.INV(RAND(),0,SQRT(LN(1+CV^2))))"

Here, State denotes the cell indicating Bim deletion mutation status, P_normal and P_delete represent the typical parameter values under non–Bim deletion and Bim deletion conditions, respectively, and CV denotes the variability of the desired log-normal distribution.

2. Supplementary Tables

Table S1. List of chemicals, reagents and cell lines used in this study.

Chemicals and reagents (Abbreviation)	Catalog	Sources
Pemetrexed Disodium Salt Heptahydrate	P303530	Aladdin (Shanghai, China)
Osimertinib Mesylate	BD295391	Bidepharm (Shanghai, China)
PC-9	SCSP-5085	Cell Bank of the Chinese Academy of Sciences (Shanghai, China)
NCI-H1650	SCSP-592	Cell Bank of the Chinese Academy of Sciences (Shanghai, China)
HCC-827	SCSP-538	Cell Bank of the Chinese Academy of Sciences (Shanghai, China)
NCI-H1975	SCSP-597	Cell Bank of the Chinese Academy of Sciences (Shanghai, China)
Pierce™ Rapid Gold BCA	A53227	Invitrogen (Carlsbad, CA, USA)
1 × PBS		KeyGen BioTech, Nanjing, China
RPMI-1640		KeyGen BioTech, Nanjing, China
Fetal Bovine Serum (FBS)		Procell, Wuhan, China
p-EGFR	3777S	Cell Signaling Technology (Danvers, MA, USA)
EGFR	4267S	Cell Signaling Technology (Danvers, MA, USA)
Bim	2933S	Cell Signaling Technology (Danvers, MA, USA)
CL-PARP	5625S	Cell Signaling Technology (Danvers, MA, USA)
PARP	9532S	Cell Signaling Technology (Danvers, MA, USA)
β-tubulin	A12289	ABclonal (Wuhan, Hubei, China)
β-Actin	20536-1-AP	Proteintech (Rosemont, IL, USA)
Formic acid **(FA)**	HPLC Grade	-
Ammonium Formate **(AF)**	HPLC Grade	-
Ammonium Acetate **(AA)**	HPLC Grade	-
Methanol (**MeOH**)	HPLC Grade	-
Acetonitrile (**ACN**)	HPLC Grade	-

Table S2. Genetic Backgrounds and characters of the Four EGFR-Mutant NSCLC Cell Lines

	**PC9**	**HCC827**	**NCI-H1975**	**NCI-H1650**
EGFR mutation	19 del [8] [1]	19 del [8]	L858R [8] [2]	19 del [9]
EGFR expression	High [3]	High [10] [3]	Low [10] [3]	Low [10] [3]
EGFR related resistance mutation	NA[4]	NA	T790M [8]	NA
EGFR sub path way related resistance mutation	NA	NA	NA	PTEN loss
Bim expression	high [3]	high [3]	low [3]	low [3]
Bim related mutation	NA	NA	NA	NA
p53 related mutation	p53-R248Q [11] [5]	p53-p.V218del [11] [6]	p53-R273H [11] [7]	p53-p.V225fs [12] [8]
Rad51 related mutation	NA	NA	NA	NA
Rad51 expression	-	Relatively low[9]	-	-
TS related mutation	The TS gene exhibits polymorphisms, but no disease-causing mutations were identified/found.			
TS expression	-	Relatively low[9]	-	-

Note: This table is an expansion version table from our previous work [13]
[1] 19del: EGFR exon 19 deletions, EGFR c.2235_2249del15 (p.Glu746_Ala750del)
[2] L858R: EGFR c.2573T>G (p.Leu858Arg), rs121434568
[3] Expression levels were inferred from the results in Figure S2.
[4] NA (not applicable): No relevant reports found
[5] p53-R248Q: TP53 c.743G>A (p.R248Q), rs11540652,
[6] p53-p.V218del: TP53 c.652_654delTGT (p.Val218del)

Table S3. Source-Gas dependent parameters of LC-MS/MS method to detect PEM

**Source-Gas dependent parameters (unit)**	**Setting**
Collision Gas (CAD) psi	6
Curtain Gas (CUR) psi	50
GS1 (psi)	65
GS2 (psi)	65
Ion Sprary (IS) V	5500
Temperature (TEM) °C	600
Interface Heater (Ihe)	ON
Entrance Potential (EP) V	10
Entrance Potential (EP) V	10

CAD: Collision Gas; **CUR:** Curtain Gas; **GS1:** ion spray gas; **GS2:** Auxiliary gas; **IS:** Ion Spary voltage; **TEM:** Temperature; **ihe:** Interface Heater; **EP:** Entrance Potential

Table S4. Compounds dependent parameters of LC-MS/MS method to detect PEM

**Compound**	**SRM transitions**	**Dwell time (msec)**	**DP (V)**	**CE (V)**	**CXP (V)**
Pemetrexed (PEM)	428.3 → 281.01	350	60	28	18
Imatinib (IMA)	494.4 → 394.1	350	80	36	11

DP: Declustering Potential**; CE:** Collision Energy**; CXP:** Collision Cell Exit Potential

Table S5. Chromatographic parameters of LC-MS/MS method to detect PEM

Chromatographic Parameters	Parameters value
Mobile phase A	0.1% formic acid in UP water
Mobile phase B	Methanol
Column	ACE C18 Amide (2.0 mm×100 mm, 3 μm)
Column temperature	40°C
Flow rate	0.3 mL/min

Table S6. Gradient Program for LC-MS/MS methods of PEM

time (min)	B%
0.1	5
4.3	55
4.5	95
5.5	95
5.7	5
7.5	5

Table S7. Standard curve setup for LC-MS/MS methods of PEM in different application scenarios

Scenario (Bio-matrix)	Concentration set up of standard curve
Cell lysates	3, 10, 30, 100, 300, 1000, 3000 nM
Plasma from NCI-H1975 NOD-SCID tumor bearing mice	0.15, 0.5, 1.5, 5, 15, 50, 150 μg/mL
Plasma from PC9 Balb/c Nude tumor bearing mice	0.15, 0.5, 1.5, 5, 15, 50, 150 μg/mL
Plasma from SD rat	0.45, 1.5, 5, 15, 50, 150, 450 μg/mL
Tumor tissue homogenates	0.15, 0.5, 1.5 , 5 ,15, 50, 150 μg/mL

Table S8. Source-Gas dependent parameters of LC-MS/MS method to detect OSI

Source-Gas dependent parameters (unit)	Setting
Collision Gas (CAD) psi	7
Curtain Gas (CUR) psi	40
GS1 (psi)	50
GS2 (psi)	50
Ion Sprary (IS) V	5000
Temperature (TEM) °C	550
Interface Heater (Ihe)	ON
Entrance Potential (EP) V	10

CAD: Collision Gas; **CUR**: Curtain Gas; **GS1**: ion spray gas; **GS2**: Auxiliary gas; **IS**: Ion Spary voltage; **TEM**: Temperature; **ihe**: Interface Heater; **EP**: Entrance Potential

Table S9. Compounds dependent parameters of LC-MS/MS method to detect OSI

	SRM transitions	Dwell time (msec)	DP (V)	CE (V)	CXP (V)
OSI	500.4 → 71.9	125	80	50	13
ERLO	394.3 → 278.2	125	90	42	18

DP: Declustering Potential**;** **CE:** Collision Energy**;** **CXP:** Collision Cell Exit Potential

Table S10. Chromatographic parameters of LC-MS/MS method to detect OSI

Chromatographic Parameters	Parameters value
Mobile phase A	0.1% formic acid and 10 mM Ammonium Acetate in UP water
Mobile phase B	0.1% Formic acid in Acetonitrile
Column	ACE C18 Super (2.0 mm×100 mm, 3 μm)
Column Temperature	40°C
Flow rate	0.5 mL/min

Table S11. Gradient Program for LC-MS/MS methods of OSI

Time (min)	Conc. B%
0.1	5
3.5	75
3.7	95
5.8	95
6	5
7	5

Table S12. Standard curve setup for LC-MS/MS methods of OSI in different application scenarios

Scenario (Bio-matrix)	Concentration set up of standard curve
Cell lysates	0.1, 0.3, 1, 3, 10, 30, 100, 300 nM
Plasma from NCI-H1975 NOD-SCID tumor bearing mice	1, 3, 10, 30, 100, 300, 1000 ng/mL
Plasma from PC9 Balb/c Nude tumor bearing mice	1, 3, 10, 30, 100, 300, 1000 ng/mL
Plasma from SD rat	1, 3, 10, 30, 100, 300, 1000 ng/mL

Table S13. Plasma Sampling Schedule for PK-DDI Studies Under Different Scenarios

Scenario (Bio-matrix)	detect	Time-points for plasma collection
Rat: PEM + OSI	PEM and OSI	5 min, 10 min, 15 min, 30 min, 45 min, 1 h, 2 h, 3h, 4 h, 6 h, 8 h, 10 h, 12 h, 24 h
Rat: OSI → PEM	PEM	5 min, 10 min, 15 min, 30 min, 45 min, 1 h, 2 h, 3 h, 4 h
PC9 Balb/c Nude	PEM	5 min, 15 min, 30 min, 45 min, 1 h, 1.5 h, 2 h, 3 h, 4 h
PC9 Balb/c Nude	OSI	5 min, 15 min, 30 min, 45 min, 1 h, 1.5 h, 2 h, 3 h, 4 h, 6 h, 8 h, 24 h
NCI-H1975 Balb/c Nude	PEM	5 min, 15 min, 30 min, 45 min, 1 h, 1.5 h, 2 h, 3 h
NCI-H1975 Nod- SCID	OSI	15 min, 30 min, 1 h, 1.5 h, 2 h, 4 h, 6 h, 8 h, 10 h
NCI-H1975 Nod-SCID	PEM	4 hr (collect tumor tissue and plasma)

Plasma collection method: Whole blood was collected into an anticoagulant-containing EP tube, centrifuged at 3000 × *g* for 10 min, and the supernatant was collected as plasma.

Table S14. The NCA parameters of PEM in rat (PEM alone group v.s. PEM + OSI group)

Subject	C_{max} (ug/ml)		AUC_{all} (h*ug/L)		Vz_obs (L)		Cl_obs (L/h)	
	PEM	PEM+OSI	PEM	PEM+OSI	PEM	PEM+OSI	PEM	PEM+OSI
1	133.00	250.00	223.30	190.51	0.22	0.15	0.10	0.12
2	315.00	306.00	239.95	209.33	0.11	0.26	0.09	0.10
3	252.00	82.60	194.67	136.74	0.15	0.33	0.11	0.16
4	362.00	348.00	209.66	274.59	0.20	0.18	0.10	0.09
5	329.00	405.00	186.70	263.82	0.12	0.24	0.13	0.08
6	297.00	293.00	189.53	200.60	0.32	0.16	0.11	0.12
Mean	281.33	280.77	207.30	212.60	0.19	0.22	0.11	0.11
RSD	28.89	39.33	10.18	23.86	41.83	32.33	12.49	23.18
Geo Mean	268.35	252.79	206.43	207.25	0.17	0.21	0.11	0.11
Geo Mean PEM+OSI/ PEM	0.94		1.00		1.22		1.03	

PEM: i.v. injection 70 mg/kg , **OSI:** oral gavage 10 mg/kg

Table S15. The NCA parameters of OSI in rat (OSI alone group v.s. PEM + OSI group)

Subject	C_{max} (ng/ml)		AUC_{all} (h*ng/mL)		Vz_F_obs (L)		Cl_F_obs (L/h)	
	OSI	PEM+OSI	OSI	PEM+OSI	OSI	PEM+OSI	OSI	PEM+OSI
1	158.00	230.00	1118.24	1875.09	14.05	7.40	2.42	1.33
2	148.00	208.00	1262.90	1976.59	11.54	7.06	2.04	1.30
3	295.00	260.00	2531.87	2560.72	7.20	4.97	1.12	1.04
4	260.00	295.00	2626.08	3234.24	5.98	5.47	1.00	0.75
5	180.00	268.00	1301.93	1920.71	10.51	7.01	2.00	1.31
6	147.00	143.00	1319.79	1412.55	10.68	13.13	2.03	1.74
Mean	198.00	234.00	1693.47	2163.32	9.99	7.51	1.77	1.25
RSD	32.16	23.03	40.76	29.56	29.53	38.93	32.34	26.33
GeoMean	190.28	228.04	1589.61	2089.73	9.60	7.12	1.68	1.21
GeoMean Ratio of PEM+OSI/OSI	1.20		1.31		0.74		0.72	

PEM: i.v. injection 70 mg/kg , **OSI:** oral gavage 10 mg/kg

Table S16. The NCA parameters of PEM in rat (1% Tween-80 8 day treated group *v.s.* OSI 10 mg/kg 8 day treated group)

**Subject**	**C_{max} (ug/ml)**		**AUC_{all} (h*ng/ml)**		**V_{z_obs} (L)**		**Cl_obs (L/h)**	
	1% tween-80 7 day	OSI 7 day	1% tween-80 7 day	OSI 7 day	1% tween-80 7 day	OSI 7 day	1% tween-80 7 day	OSI 7 day
1	294.00	334.00	222368.17	208832.42	0.11	0.10	0.11	0.11
2	401.00	411.00	198269.78	213462.28	0.09	0.08	0.13	0.11
3	293.00	361.00	184678.67	191562.75	0.10	0.12	0.13	0.13
4	361.00	363.00	172067.96	188506.74	0.08	0.08	0.13	0.12
5	293.00	396.00	166102.58	211053.51	0.10	0.08	0.16	0.12
6	327.00	407.00	146952.18	228170.62	0.09	0.08	0.17	0.11
Mean	328.17	378.67	181739.89	206931.39	0.10	0.09	0.14	0.12
RSD	13.64	8.10	14.52	7.14	13.10	18.83	15.78	6.83
GeoMean	325.75	377.61	180163.32	206491.20	0.09	0.09	0.14	0.12
OSI 7 day/tween-80 7 day	1.16		1.15		0.96		0.87	

PEM: i.v. injection 70 mg/kg , **OSI:** oral gavage 10 mg/kg

Talbe S17 The detailed differential equations (ODEs) in the QSP-PK-PD model for the combination of PEM and OSI.

No.	ODEs
1	d(X1)/dt = 1/[Tumor Compartment]*(((Lambda_0*X1/(1+(Lambda_0*X1/Lambda_1)^psi)^(1/psi))*EGFR^gamma_EGFR) - (k1*X1*(1+Emax_folate*(1-Folate)^gamma_folate/(EC50_folate^gamma_folate+(1-Folate)^gamma_folate))*EGFR^gamma_G1))
2	d(X2)/dt = 1/[Tumor Compartment]*((k1*X1*(1+Emax_folate*(1-Folate)^gamma_folate/(EC50_folate^gamma_folate+(1-Folate)^gamma_folate))*EGFR^gamma_G1) - (k2*X2*(1+k_bim*(1-EGFR)^gamma_bim)))
3	d(X3)/dt = 1/[Tumor Compartment]*((k2*X2*(1+k_bim*(1-EGFR)^gamma_bim)) - (k2*X3*(1+k_bim*(1-EGFR)^gamma_bim)))
4	d(X4)/dt = 1/[Tumor Compartment]*((k2*X3*(1+k_bim*(1-EGFR)^gamma_bim)) - (k2*X4*(1+k_bim*(1-EGFR)^gamma_bim)))
5	d(X1_unperturbed)/dt = 1/[Tumor Compartment]*(((Lambda_0*X1_unperturbed/(1+(Lambda_0*X1_unperturbed/Lambda_1)^psi)^(1/psi))) - (k1*X1_unperturbed))
6	d(X2_unperturbed)/dt = 1/[Tumor Compartment]*((k1*X1_unperturbed) - (k2*X2_unperturbed))
7	d(X3_unperturbed)/dt = 1/[Tumor Compartment]*((k2*X2_unperturbed) - (k2*X3_unperturbed))
8	d(X4_unperturbed)/dt = 1/[Tumor Compartment]*((k2*X3_unperturbed) - (k2*X4_unperturbed))
9	d(total_Death)/dt = 1/[Tumor Compartment]*((k2*X4*(1+k_bim*(1-EGFR)^gamma_bim)))
10	d(PEM_1)/dt = 1/PEM_V1*(-((k12_pem*PEM_1-k21_pem*PEM_2)*PEM_V1) + ((ka_pem*PEM_A)*PEM_V1) - ((kel_pem*PEM_1)*PEM_V1))
11	d(PEM_A)/dt = 1/PEM_V1*(-((ka_pem*PEM_A)*PEM_V1))
12	d(PEM_2)/dt = 1/PEM_V2*(((k12_pem*PEM_1-k21_pem*PEM_2)*PEM_V1))
13	d(Folate)/dt = 1/Folate_1*((kout_folate*Enzyme^gamma_Enzyme) - (kout_folate*Folate))
14	d(Enzyme)/dt = 1/Folate_1*((kout_Enzyme) - (kout_Enzyme*Enzyme*(1+Emax_pem*PEM_1^gamma_pem/(EC50_pem^gamma_pem+PEM_1^gamma_pem))))
15	d(OSI_1)/dt = 1/OSI_V1*(-((k12_osi*OSI_1)*OSI_V1-(k21_osi*OSI_2)*OSI_V2) + ((ka_osi*OSI_A)*OSI_V1) - ((k_el_osi*OSI_1)*OSI_V1))
16	d(OSI_A)/dt = 1/OSI_V1*(-((ka_osi*OSI_A)*OSI_V1))
17	d(OSI_2)/dt = 1/OSI_V2*(((k12_osi*OSI_1)*OSI_V1-(k21_osi*OSI_2)*OSI_V2))
18	d(EGFR)/dt = 1/EGFR_1*((kout_EGFR*(1+k_EGFR_feedback*Damaged_percent^gamma_EGFR_feedback)) - (kout_EGFR*EGFR*(1+Imax_osi*OSI_1^gamma_osi/(EC50_osi^gamma_osi+OSI_1^gamma_osi))))

Note: gamma_pem = 1, and gamma_folate = 1 (gamma_pem and gamma_folate are not included in this model)

Talbe S18 The detailed algebraic relationships for the special compartment

No.	Compartment to be defined	Algebraic relationships
1	X_total	X_total = X1+X2+X3+X4
2	X_damged	X_damaged = X2+X3+X4
3	damaged_percent	Damaged_percent = X_damaged/X_total
4	X_unperturbed_total	X_uperturbed_total = X1_uperturbed+X2_uperturbed+X3_uperturbed+X4_uperturbed
5	TGI	TGI=1-X_total/X_total_unperturbed
6	X1_percent	X1_percent=X1/X_total

Table S19 The values and descriptions of the model parameters in the QSP-PK-PD model for the PEM–OSI combination. 3

Module	Parameters	Description	Source	Value	Unit	Values in literature	Reference (doi)
TGI Module	Lambda_0	Exponential growth rate constant	Curve Fitting	0.1032	1/day	0.1	10.1124/jpet.118.248286
	Lambda_1	Linear phase growth rate constant	Curve Fitting	51.08	mm^3/day	35.1	10.1124/jpet.118.248286
	psi	Power term governing the transition sharpness	Literature	20	Dimensionless	20	10.1158/0008-5472.can-03-2524
	k1	Natural rate for transition proliferating cells to damaged tumor cells	Curve Fitting	0.016	1/day	0.0218	10.1124/jpet.117.243931
	k2	Natural apoptosis rate in damaged tumor cells	Curve Fitting	0.0045	1/day		
	gamma_bim	Power term on k_bim	IVIVE	0.595	Dimensionless		
	gamma_G1	Power term of EGFR level's impact on the cytoxicity of k1	Assumed Value	8	Dimensionless		
	gamma_EGFR	Power term of EGFR level's impact on the proliferation rate of X1 compartment	IVIVE	3.998	Dimensionless		
	Emax_folate	Maximum fractional increase in k1 due to folate level decrease	IVIVE	49.5	Dimensionless		
	EC50_folate	EC50 value for Emax_folate (hill function)	Assumed Value	0.5	Dimensionless		
	k_bim	Maximum fractional increase in k2 due to EGFR level decrease	Literature	211	Dimensionless	211	10.1158/1535-7163.MCT-16-0142 (N_PC9, also means f_kill)

Folate Module	kout_Enzyme	Turn over rate for folate metaboling relative enzyme	Assumed Value	1	1/day		
	kout_folate	Turn over rate for folate	Assumed Value	3	1/day		
	gamma_Enzyme	Power term of Enzyme level's impact on kin of folate	IVIVE	2.0129	Dimensionless		
	Emax_pem	PEM drived maximum fractional increase in kout,enzyme	IVIVE	5.56	Dimensionless		
	EC50_pem,medium	EC50 value for Emax_pem (Hill function) im medium	in vitro data	300	nM		
	EC50_pem, plasma	EC50 value for Emax_pem (Hill function)	IVIVE	0.47315	mg/L		
EGFR Module	kout_EGFR	Turn over rate for EGFR signal	IVIVE	1.5	1/day	1.44,1.3, 1.9	10.1158/1535-7163.mct-16-0142(1.44, based on krec_a431), 10.1038/aps.2013.101（1.30）10.1038/aps.2016.40 (1.9)
	k_EGFR_feedback	Damaged tumor cells drived fractional increase in kin,EGFR	Curve Fitting	0.2	Dimensionless	-	
	gamma_EGFR_feedback	Power term on k_EGFR_feedback	Curve Fitting	1.45	Dimensionless	-	
	Imax_osi	Binding rate of drug to EGFR	Literature	22.872	Dimensionless	22.872/day (0.953/h)	10.1158/1535-7163.MCT-16-0142 (K_bind and $I_{max,osi}$ are equal means, represent binding rate of osi to EGFR)
	EC50_osi_medium	EC50 value for Imax_osi (hill function) in medium	in vitro data	14.49	nM	17 and 15	10.1158/1535-7163.MCT-16-0142
	EC50_osi_plasma	EC50 value for Imax_osi (hill function) in plasma	IVIVE	48.866	μg/L	84.93523	10.1158/1535-7163.MCT-22-0193, Calculated as MW * EC50, MW_{osi} = 499.619, $EC_{50,osi,plasma}$ = 0.17 μM

	gamma_osi	Power term on EC50,osi,plasma	Assumed Value	2	Dimensionless		
PEM PK Module	ka_pem	Absorption rate of PEM following intraperitoneal injection	PK Analysis	102.3379	1/day		
	kel_pem	Elimination rate of PEM from central compartment	PK Analysis	56.6998	1/day	51.1872	10.1002/psp4.12265 (Supplementary Code, transformed the value from per hr to per day)
	k12_pem	translation rate of PEM form central compartment to peripheral compartment	PK Analysis	2.3398	1/day		
	k21_pem	translation rate of PEM form peripheral compartment to central compartment	PK Analysis	2.8223	1/day		
	PEM_V1	Apparent volume of PEM in central compartment	PK Analysis	0.39125	L/kg		
	PEM_V2	Apparent volume of PEM in peripheral compartment	PK Analysis	0.32436	L/kg		
OSI PK Module	ka_osi	Absorption rate of OSI following oral gavage	PK Analysis	24.6514	1/day		
	k_el_osi	Elimination rate of OSI from central compartment	PK Analysis	10.6071	1/day	12.18660287	10.1158/1535-7163.MCT-16-0142 (Supplementary Material Calculated through k = $CL_p/V_{1,p}$
	k12_osi	translation rate of OSI form central compartment to peripheral compartment	PK Analysis	11.005	1/day		
	k21_osi	translation rate of OSI form peripheral compartment to central compartment	PK Analysis	5.4194	1/day		
	OSI_V1/F	Apparent volume of OSI in central compartment	PK Analysis	7.1758	L/kg	4.495	10.1158/1535-7163.MCT-16-0142 (Supplementary Material $V_{ss,p}$)
	OSI_V2/F	Apparent volume of OSI in peripheral compartment	PK Analysis	14.5716	L/kg		

Table S20 The initial values in each compartment in the QSP-PK-PD model for the PEM–OSI combination.

Module	Compartment	Description	Unit	initial Value	Algebraic relationships
TGI Module	X1	Proliferating Tumor cells compartment	mm^3	200	
	X2	Damaged tumor cells compartment (series 1)	mm^3	0	
	X3	Damaged tumor cells compartment (series 2)	mm^3	0	
	X4	Damaged tumor cells compartment (series 3)	mm^3	0	
	X_total	Total tumor volume	mm^3	200	X_total = X1+X2+X3+X4
	X_damaged	Damaged tumor volume	mm^3	0	X_damaged = X2+X3+X4
	Damaged_percent	percent of damaged tumor volume on the total tumor volume	unitless	0	Damaged_percent = X_damaged/X_total
	TGI	inhibition rate of X_total relative to X_total_unperturbed	unitless	0	TGI=1-X_total/X_unperturbed_total
	total_Death	Total tumor volume that eliminated from X4	mm^3	0	
	X1_unperturbed	Proliferating Tumor cells compartment (with out drug treated)	mm^3	200	
	X2_unperturbed	Damaged tumor cells compartment (series 1) (with out drug treated)	mm^3	0	
	X3_unperturbed	Damaged tumor cells compartment (series 2) (with out drug treated)	mm^3	0	
	X4_unperturbed	Damaged tumor cells compartment (series 3) (with out drug treated)	mm^3	0	
	X_unperturbed_total	Total tumor volume (with out drug treated)	mm^3	200	X_unperturbed_total=X1_unperturbed+X2_unperturbed+X3_unperturbed+X4_unperturbed
Folate Module	Folate	Folate level compartment	unitless	1	
	Enzyme	Folate metabolism related enzyme level compartment	unitless	1	
EGFR Module	EGFR	EGFR level compartment	unitless	1	
PEM PK Module	PEM_A	PEM Absorption Compartment	mg/L	0	
	PEM_1	PEM Central Compartment	mg/L	0	
	PEM_2	PEM peripheral Compartment	mg/L	0	
OSI PK Module	OSI_A	OSI Absorption Compartment	μg/L	0	
	OSI_1	OSICentral Compartment	μg/L	0	
	OSI_2	OSI peripheral Compartment	μg/L	0	

Table S21 The detailed reaction scheme in the QSP-PK-PD model for the PEM–OSI combination.

No.	Reaction Scheme
1	null -> [Tumor Compartment].X1<br>(Lambda_0*X1/(1+(Lambda_0*X1/Lambda_1)^psi)^(1/psi))*EGFR ^ gamma_EGFR
2	[Tumor Compartment].X1 -> [Tumor Compartment].X2<br>k1*[Tumor Compartment].X1 * (1 + Emax_folate * (1-Folate) ^ gamma_folate / (EC50_folate ^ gamma_folate + (1-Folate) ^ gamma_folate)) * EGFR^gamma_G1
3	[Tumor Compartment].X2 -> [Tumor Compartment].X3<br>k2*[Tumor Compartment].X2 * (1+k_bim *(1-EGFR)^gamma_bim)
4	[Tumor Compartment].X3 -> [Tumor Compartment].X4<br>k2*[Tumor Compartment].X3 * (1+k_bim *(1-EGFR) ^gamma_bim)
5	[Tumor Compartment].X4 -> null<br>k2*[Tumor Compartment].X4 * (1+k_bim *(1-EGFR)^gamma_bim)
6	PEM_V1.PEM_1 <-> PEM_V2.PEM_2<br>k12_pem*PEM_V1.PEM_1 - k21_pem*PEM_V2.PEM_2
7	PEM_V1.PEM_A -> PEM_V1.PEM_1<br>ka_pem*PEM_V1.PEM_A
8	PEM_V1.PEM_1 -> null<br>kel_pem*PEM_V1.PEM_1
9	null -> Folate_1.Folate<br>kout_folate*Enzyme^gamma_Enzyme
10	Folate_1.Folate -> null<br>kout_folate*Folate_1.Folate
11	OSI_V1.OSI_1 <-> OSI_V2.OSI_2<br>k12_osi*OSI_V1.OSI_1 - k21_osi*OSI_V2.OSI_2
12	OSI_V1.OSI_A -> OSI_V1.OSI_1<br>ka_osi*OSI_V1.OSI_A
13	OSI_V1.OSI_1 -> null<br>k_el_osi*OSI_V1.OSI_1
14	null -> Folate_1.Enzyme<br>kout_Enzyme
15	Folate_1.Enzyme -> null<br>kout_Enzyme*Folate_1.Enzyme*(1+Emax_pem*PEM_V1.PEM_1^gamma_pem/(EC50_pem^gamma_pem+PEM_V1.PEM_1^gamma_pem))
16	null -> EGFR_1.EGFR<br>kout_EGFR*(1+k_EGFR_feedback*Damaged_percent^gamma_EGFR_feedback)
17	EGFR_1.EGFR -> null<br>kout_EGFR*EGFR_1.EGFR*(1+Imax_osi*OSI_1^gamma_osi/(EC50_osi^gamma_osi+OSI_1^gamma_osi))
18	null -> [Tumor Compartment].X1_unperturbed<br>(Lambda_0*X1_unperturbed/(1+(Lambda_0*X1_unperturbed/Lambda_1)^psi)^(1/psi))
19	[Tumor Compartment].X1_unperturbed -> [Tumor Compartment].X2_unperturbed<br>k1*[Tumor Compartment].X1_unperturbed
20	[Tumor Compartment].X2_unperturbed -> [Tumor Compartment].X3_unperturbed<br>k2*[Tumor Compartment].X2_unperturbed
21	[Tumor Compartment].X3_unperturbed -> [Tumor Compartment].X4_unperturbed<br>k2*[Tumor Compartment].X3_unperturbed

22	[Tumor Compartment].X4_unperturbed -> null<br>k2*[Tumor Compartment].X4_unperturbed
23	null -> [Tumor Compartment].total_Death<br>k2*[Tumor Compartment].X4 * (1+k_bim *(1-EGFR)^gamma_bim)

Note: gamma_pem = 1, and gamma_folate = 1 (gamma_pem and gamma_folate are not included in this model)

3. Supplementary Figures

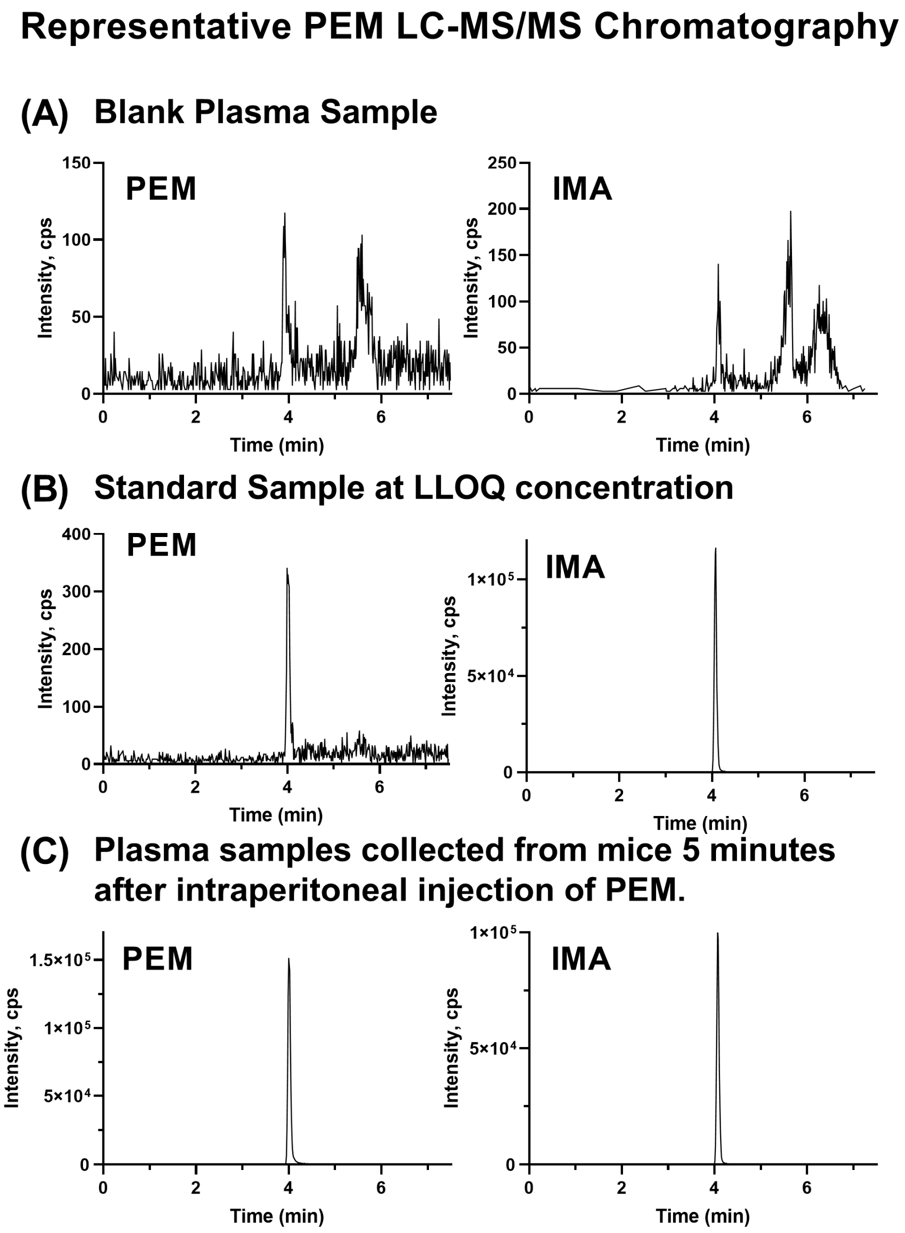

Figure S1. Representative PEM LC-MS/MS chromatography and its internal standard (imatinib, IMA) of: (A) blank plasma sample; (B) LLOQ sample (0.15 μg/mL); (C) Plasma samples collected from PC-9 tumor bearing Balb/C nude mice 5 minutes after intraperitoneal injection of PEM (100 mg/kg)

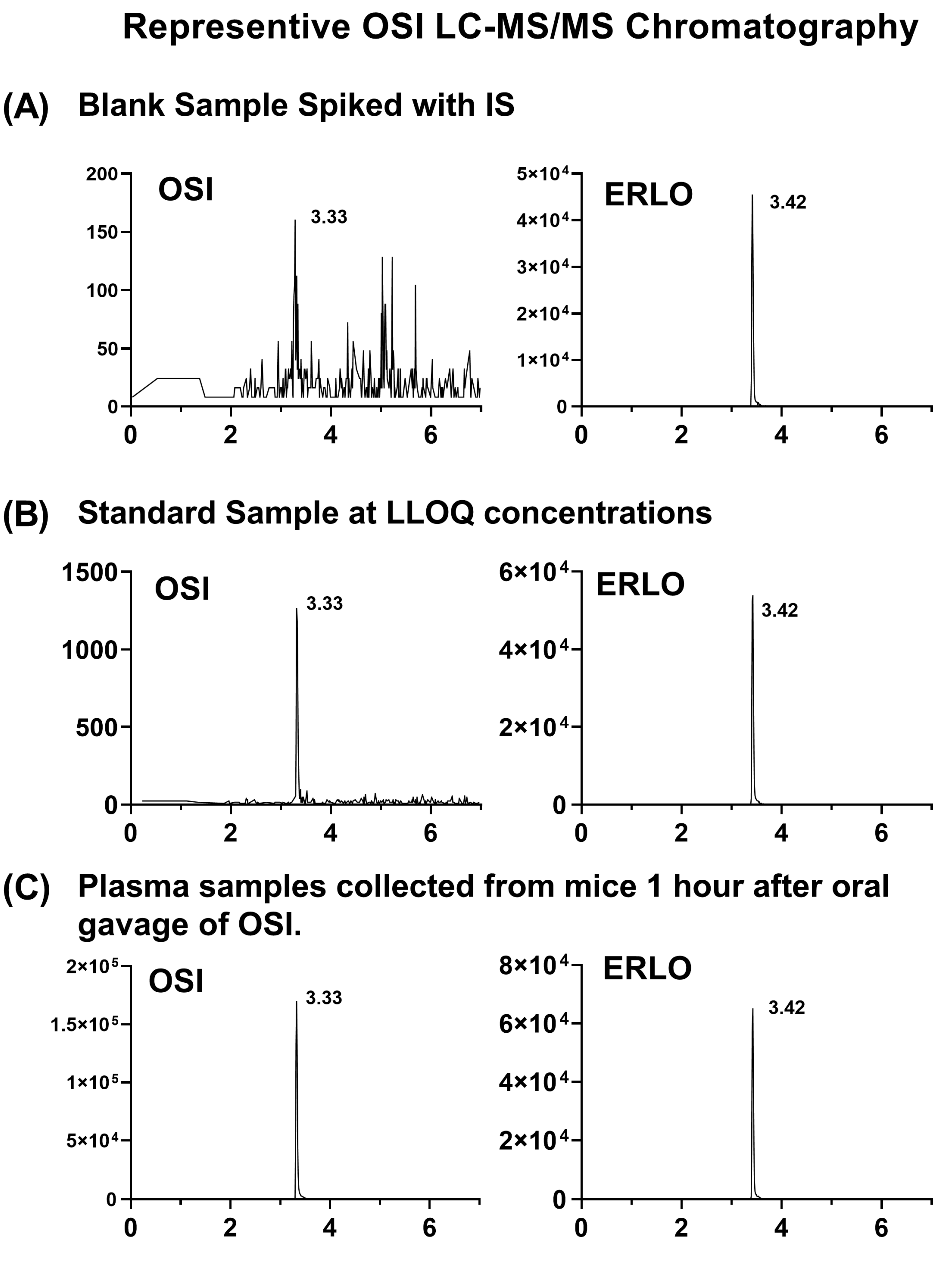

Figure S2. Representative OSI LC-MS/MS chromatography and its internal standard (erlotinib, ERLO) of: (A) blank plasma sample spiked with IS; (B) LLOQ sample (5 ng/mL); (C) Plasma samples collected from PC-9 tumor bearing Balb/C nude mice 1 hour after oral gavage of OSI (5 mg/kg).

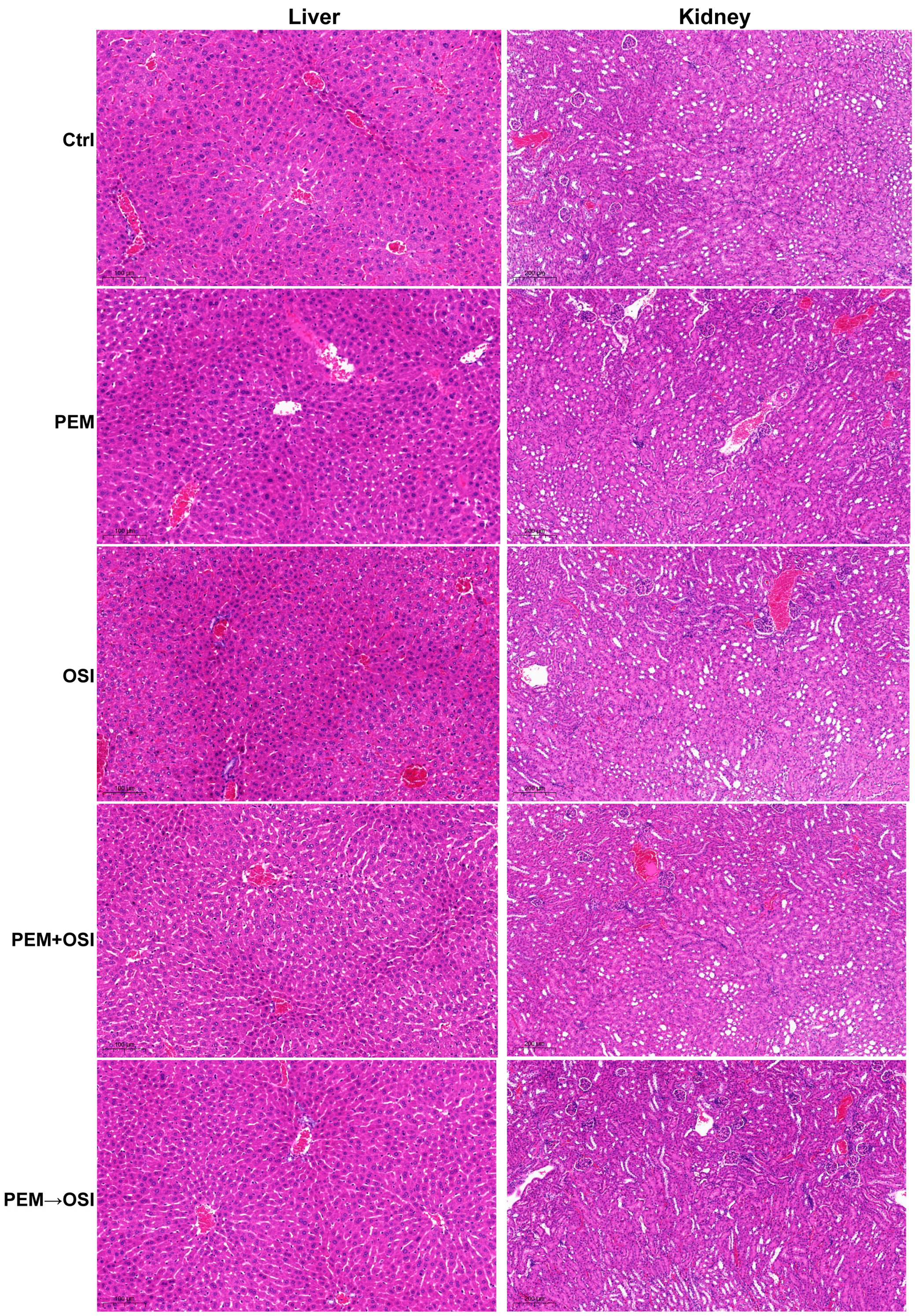

Figure S3. Safety evaluation of PEM (105 mg/kg/day) and OSI (1 mg/kg) monotherapy and their combinations under different combination strategies. The figure presents 20x histological sections of liver and kidney tissues across different groups.

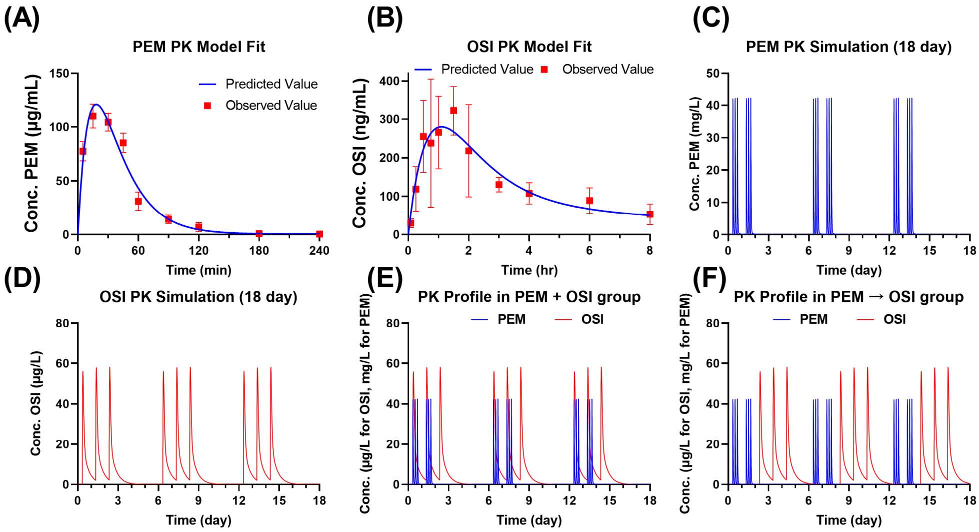

Figure S4. PK modeling and Simulation of PEM and OSI in Control, Monotherapy, and Combination Treatment Groups Under Different Dosing Strategies (As illustrated in Figure 6A). (A, B): PK models for PEM and OSI, each described by a two-compartment model. The red symbols represents observed concentrations while the blue line represent the simulated concentrations. (C-F): Simulated plasma concentration-time profiles for PEM monotherapy (C), OSI monotherapy (D), concurrent coadministration of PEM + OSI (E), and sequential administration of PEM followed by OSI (PEM → OSI) (F). All data are presented as Mean ± SD.

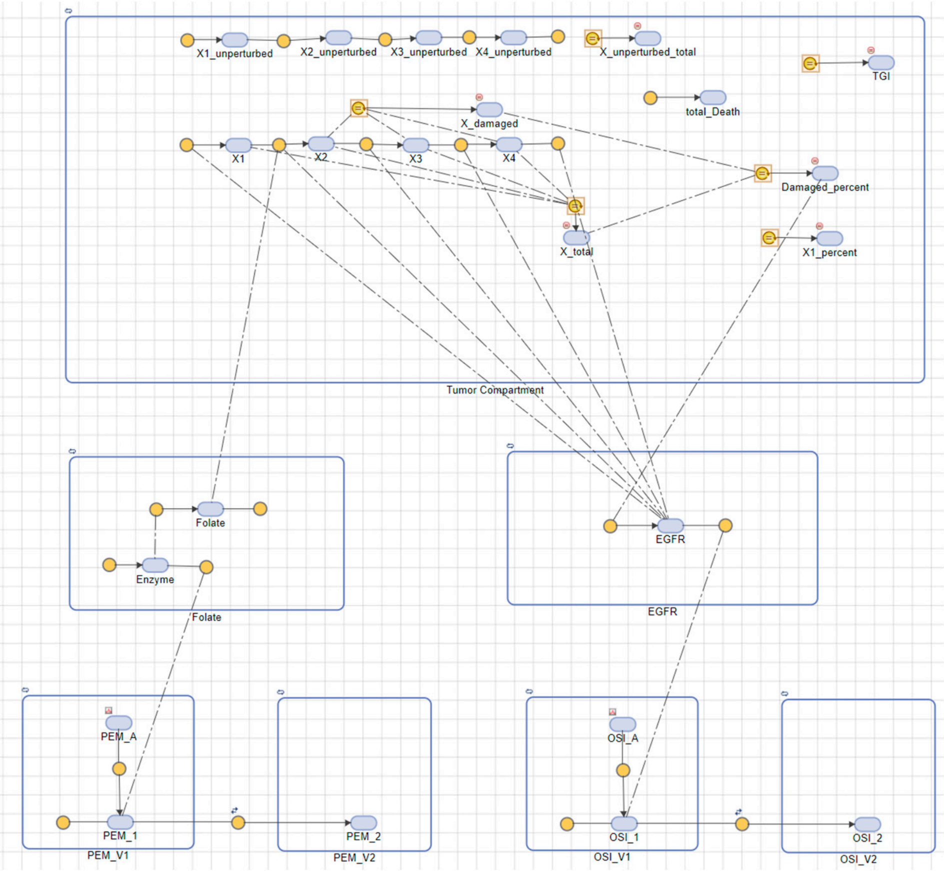

Figure S5. The overall QSP-PK-PD model structure as presented in the Sim-Biology software.

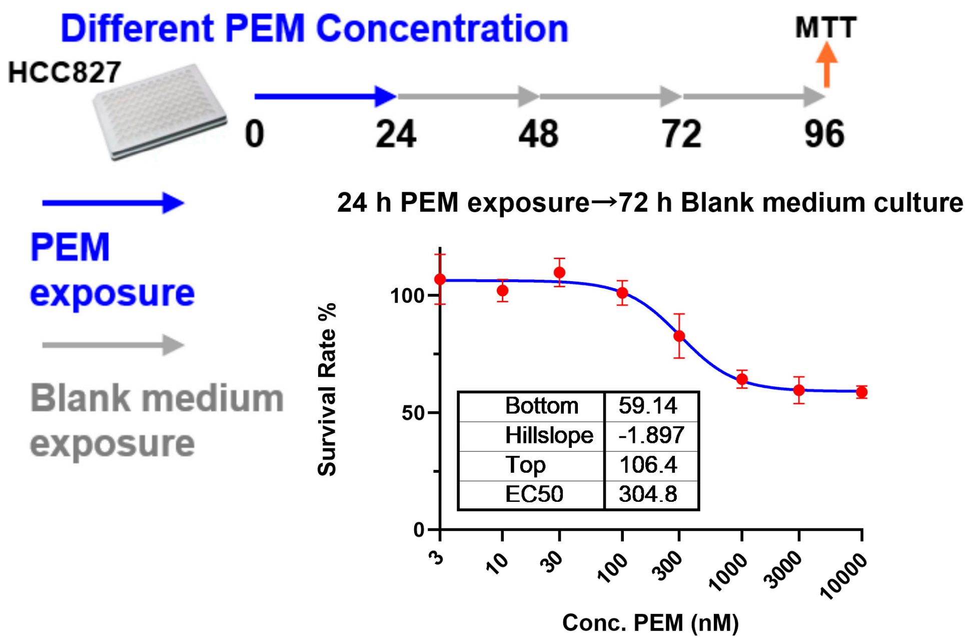

Figure S6. Schematic diagram illustrating the *in vitro* estimation of the $EC_{50,pem,medium}$.

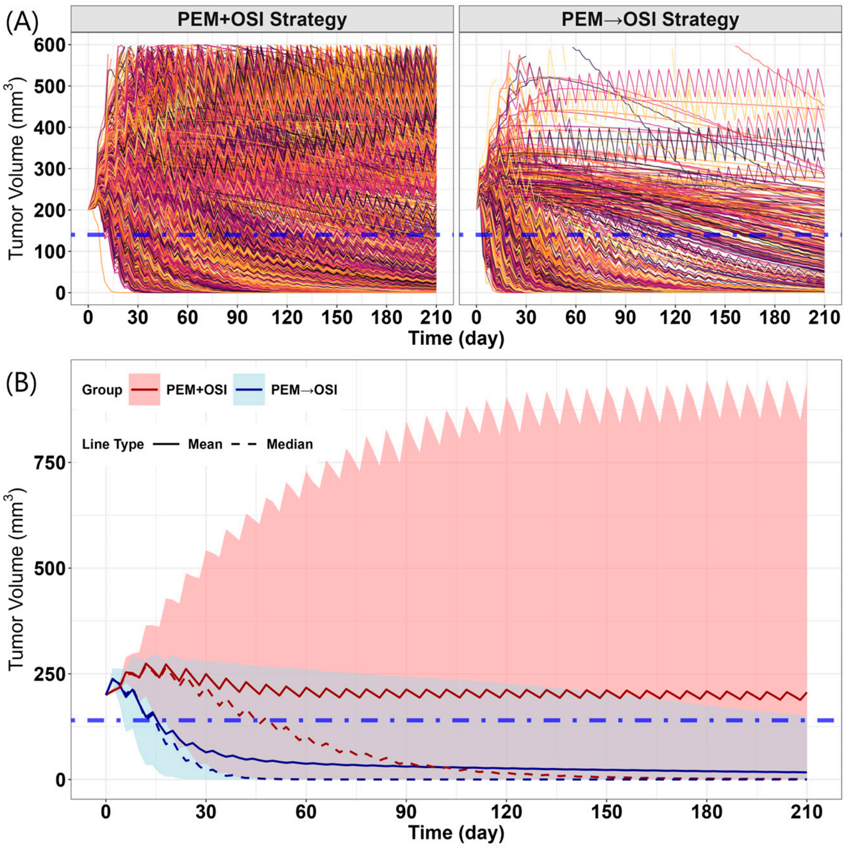

Figure S7. Monte Carlo Simulation of PEM→OSI and PEM+OSI strategies following a 210-day simulation. (A) Simulated tumor growth curves of 2000 virtual mice over 210 days. **(B)** The simulated mean, median, and 5th to 95th percentile region. The blue dashed reference line represents 70% of the initial tumor volume.

GENETIC TESTING BIOTECHNOLOGY

Cell Line Authentication Service
STR Profile Report

Sales Order: 230510B

	Test Results for Submitted Sample			ExPASy Reference Database Profile	
**Loci**	**Query Profile:** HCC827			**Database Profile:** HCC827	
Amelogenin	X			X	
D3S1358	17				
D5S818	12			12	
D2S1338	17	24			
TPOX	8			8	
CSF1PO	11			11	
Penta D	14				
TH01	6			6	
vWA	18			18	
D7S820	11	12		11	12
D21S11	31				
Penta E	20				
D10S1248	13	17			
D8S1179	12				
D1S1656	12	17	18		
D18S51	13				
D12S391	17				
D6S1043	11	12			
D19S433	14				
D16S539	12			12	
D13S317	9			9	
FGA	22	24			

The allele match algorithm compares the 8 core loci plus amelogenin only, even though alleles from all loci will be reported when available.

Note: Loci highlighted in grey (8 core STR loci plus Amelogenin) can be made public to verify cell identity. In order to protect the identity of the donor, ***please do not publish*** *the allele calls from all the STR loci tested.*

The sample match is based on the reference data available at the time of comparison.

Explanation of Test Results

Cell lines with ≥80% match are considered to be related; i.e., derived from a common ancestry. Cell lines with between a 55% to 80% match require further profiling for authentication of relatedness.

☐ The submitted sample profile is human, but not a match for any profile in the ExPASy STR database.

☑ The submitted profile is an exact match for the following human cell line(s) in the ExPASy STR database (8 core loci plus Amelogenin): HCC827

☐ The submitted profile is similar to the following ExPASy human cell line(s):

e-Signature Technician: Digitally signed by Xuekun Chen
DN: cn=Xuekun Chen, o=Genetic Testing Biotechnology (Suzhou), ou=DNA Typing Section, email=order@jsdna.org, c=CN
Date: 2023.05.10 15:38:59 +08'00'

e-Signature Reviewer: Digitally signed by Xiankun Zhao
DN: cn=Xiankun Zhao, o=Genetic Testing Biotechnology (Suzhou), ou=Supervision Section, email=service@jsdna.org, c=CN
Date: 2023.05.10 15:39:29 +08'00'

CELL LINE AUTHENTICATION

More information

Addendum: Electropherogram for the customer's sample set 1 of 1

For Research Use ONLY   Page 2 of 3   Ver. 3.1.2

Figure S8. STR certification for HCC827

GENETIC TESTING BIOTECHNOLOGY

Cell Line Authentication Service
STR Profile Report

Sales Order: 230508A

	Test Results for Submitted Sample		ExPASy Reference Database Profile	
**Loci**	**Query Profile:** PC-9		**Database Profile:** PC-9	
Amelogenin	X		X	
D3S1358	16			
D5S818	11		11	
D2S1338	19	20		
TPOX	11		11	
CSF1PO	11		11	
Penta D	9	13		
TH01	7		7	
vWA	17		17	
D7S820	10	11	10	11
D21S11	29	30		
Penta E	11	15		
D10S1248	13			
D8S1179	11	15		
D1S1656	15	18.3		
D18S51	15			
D12S391	18			
D6S1043	13	19		
D19S433	13	15.2		
D16S539	9		9	
D13S317	8		8	
FGA	23			

The allele match algorithm compares the 8 core loci plus amelogenin only, even though alleles from all loci will be reported when available.
Note: Loci highlighted in grey (8 core STR loci plus Amelogenin) can be made public to verify cell identity. In order to protect the identity of the donor, ***please do not publish*** *the allele calls from all the STR loci tested.*
The sample match is based on the reference data available at the time of comparison.

Explanation of Test Results
Cell lines with ≥80% match are considered to be related; i.e., derived from a common ancestry. Cell lines with between a 55% to 80% match require further profiling for authentication of relatedness.

☐ The submitted sample profile is human, but not a match for any profile in the ExPASy STR database.

☑ The submitted profile is an exact match for the following human cell line(s) in the ExPASy STR database (8 core loci plus Amelogenin): PC-9

☐ The submitted profile is similar to the following ExPASy human cell line(s):

e-Signature-Technician: Digitally signed by Xuekun Chen
DN: cn=Xuekun Chen, o=Genetic Testing Biotechnology (Suzhou), ou=DNA Typing Section, email=order@jsdna.org, c=CN
Date: 2023.05.08 15:35:08 +08'00'

e-Signature-Reviewer: Digitally signed by Xiankun Zhao
DN: cn=Xiankun Zhao, o=Genetic Testing Biotechnology (Suzhou), ou=Supervision Section, email=service@jsdna.org, c=CN
Date: 2023.05.08 15:35:43 +08'00'

CELL LINE AUTHENTICATION

More information

Addendum: Electropherogram for the customer's sample set 1 of 1

For Research Use ONLY — Page 2 of 3 — Ver. 3.1.2

Figure S9. STR certification for PC-9

GENETIC TESTING BIOTECHNOLOGY

Cell Line Authentication Service
STR Profile Report

Sales Order: 210817C

Loci	Test Results for Submitted Sample<br>Query Profile: NCI-H1975		DSMZ Reference Database Profile<br>Database Profile: NCI-H1975	
Amelogenin	X		X	
D3S1358	14	15		
D13S317	10	13	10	13
D7S820	8	11	8	11
D16S539	9	12	9	12
Penta E	12	16		
TPOX	8	11	8	11
TH01	7		7	
D2S1338	17			
CSF1PO	12		12	
Penta D	12	13		
D19S433	15	15.2		
vWA	18		18	
D21S11	28			
D18S51	13			
D6S1043	12			
D8S1179	13	16		
D5S818	11	12	11	12
D12S391	17			
FGA	21	24		

The allele match algorithm compares the 8 core loci plus amelogenin only, even though alleles from all loci will be reported when available.

Note: Loci highlighted in grey (8 core STR loci plus Amelogenin) can be made public to verify cell identity. In order to protect the identity of the donor, ***please do not publish*** *the allele calls from all the STR loci tested. The sample match is based on the reference data available at the time of comparison.*

Explanation of Test Results

Cell lines with ≥80% match are considered to be related; i.e., derived from a common ancestry. Cell lines with between a 55% to 80% match require further profiling for authentication of relatedness.

☐ The submitted sample profile is human, but not a match for any profile in the DSMZ STR database.

☑ The submitted profile is an exact match for the following human cell line(s) in the DSMZ STR database (8 core loci plus Amelogenin): NCI-H1975

☐ The submitted profile is similar to the following DSMZ human cell line(s):

e-Signature-Technician: Digitally signed by Xuekun Chen
DN: cn=Xuekun Chen, o=Genetic Testing Biotechnology (Suzhou), ou=DNA Typing Section, email=order@jsdna.org, c=CN
Date: 2021.08.17 16:20:37 +08'00'

CELL LINE AUTHENTICATION

e-Signature-Reviewer: Digitally signed by Xiankun Zhao
DN: cn=Xiankun Zhao, o=Genetic Testing Biotechnology (Suzhou), ou=Supervision Section, email=service@jsdna.org, c=CN
Date: 2021.08.17 16:21:11 +08'00'

Addendum: Electropherogram for the customer's sample set 1 of 1

For Research Use ONLY   Page 2 of 3   Ver. 3.1.2

Figure S10. STR certification for NCI-H1975

References

1. Ao, Luyao, Shencun Fang, Kexin Zhang, Yang Gao, Jiawen Cui, Wenjing Jia, Yunlong Shan, Jingwei Zhang, Guangji Wang, Jiali Liu, and Fang Zhou. "Sequence-Dependent Synergistic Effect of Aumolertinib-Pemetrexed Combined Therapy on Egfr-Mutant Non-Small-Cell Lung Carcinoma with Pre-Clinical and Clinical Evidence." Journal of Experimental & Clinical Cancer Research 41, no. 1 (2022).
2. La Monica, Silvia, Denise Madeddu, Marcello Tiseo, Valentina Vivo, Maricla Galetti, Daniele Cretella, Mara Bonelli, Claudia Fumarola, Andrea Cavazzoni, Angela Falco, Andrea Gervasi, Costanza Annamaria Lagrasta, Nadia Naldi, Elisabetta Barocelli, Andrea Ardizzoni, Federico Quaini, Pier Giorgio Petronini, and Roberta Alfieri. "Combination of Gefitinib and Pemetrexed Prevents the Acquisition of Tki Resistance in Nsclc Cell Lines Carrying Egfr- Activating Mutation." Journal of Thoracic Oncology 11, no. 7 (2016): 1051-63.

3. Liu, Tianze, Lizi Jin, Wenjing Lu, Hairun Gan, Zhidong Lin, Miao Chen, Jiani Liu, Fan Zhang, Siyang Wang, Hongyu Zhang, Wuguo Deng, and Hongtao Chen. "Sequence-Dependent Synergistic Cytotoxicity of Icotinib and Pemetrexed in Human Lung Cancer Cell Lines in Vitro and in Vivo." Journal of Experimental & Clinical Cancer Research 38, no. 1 (2019).
4. Kleczko, Emily K., Anh T. Le, Trista K. Hinz, Teresa T. Nguyen, Andre Navarro, Cheng-Jun Hu, Ana M. Selman, Eric T. Clambey, Daniel T. Merrick, Sizhao Lu, Mary Weiser-Evans, Raphael A. Nemenoff, and Lynn E. Heasley. "Novel Egfr-Mutant Mouse Models of Lung Adenocarcinoma Reveal Adaptive Immunity Requirement for Durable Osimertinib Response." Cancer Letters 556 (2023).
5. Cross, Darren A. E., Susan E. Ashton, Serban Ghiorghiu, Cath Eberlein, Caroline A. Nebhan, Paula J. Spitzler, Jonathon P. Orme, M. Raymond V. Finlay, Richard A. Ward, Martine J. Mellor, Gareth Hughes, Amar Rahi, Vivien N. Jacobs, Monica Red Brewer, Eiki Ichihara, Jing Sun, Hailing Jin, Peter Ballard, Katherine Al-Kadhimi, Rachel Rowlinson, Teresa Klinowska, Graham H. P. Richmond, Mireille Cantarini, Dong-Wan Kim, Malcolm R. Ranson, and William Pao. "Azd9291, an Irreversible Egfr Tki, Overcomes T790m-Mediated Resistance to Egfr Inhibitors in Lung Cancer." Cancer Discovery 4, no. 9 (2014): 1046-61.
6. Cao, Peng, Wei Guo, Jun Wang, Sanlan Wu, Yifei Huang, Yang Wang, Yani Liu, and Yu Zhang. "Population Pharmacokinetic Study of Pemetrexed in Chinese Primary Advanced Non-Small Cell Lung Carcinoma Patients." Frontiers in Pharmacology 13 (2022).
7. Yang, Jincheng, Damilola Olabode, Aarti Sawant-Basak, Richard Baldry, Karthick Vishwanathan, Srinivas Bachina, Alexandar Todd, Dana Ghiorghiu, Yuri Rukazenkov, Diansong Zhou, and Azar Shahraz. "Population Pharmacokinetics and Exposure-Response Analysis of First-Line Osimertinib Plus Chemotherapy in Patients with Egfr-Mutated Advanced Nsclc." Clinical Pharmacology & Therapeutics (2025).
8. Tanaka, Kosuke, Helena A. Yu, Shaoyuan Yang, Song Han, S. Duygu Selcuklu, Kwanghee Kim, Shriram Ramani, Yogesh Tengarai Ganesan, Allison Moyer, Sonali Sinha, Yuchen Xie, Kota Ishizawa, Hatice U. Osmanbeyoglu, Yang Lyu, Nitin Roper, Udayan Guha, Charles M. Rudin, Mark G. Kris, James J. Hsieh, and Emily H. Cheng. "Targeting Aurora B Kinase Prevents and Overcomes Resistance to Egfr Inhibitors in Lung Cancer by Enhancing Bim- and Puma-Mediated Apoptosis." Cancer Cell 39, no. 9 (2021): 1245-61.e6.
9. Ayestaran, Iñigo, Ana Galhoz, Elmar Spiegel, Ben Sidders, Jonathan R. Dry, Frank Dondelinger, Andreas Bender, Ultan McDermott, Francesco Iorio, and Michael P. Menden. "Identification of Intrinsic Drug Resistance and Its Biomarkers in High-Throughput Pharmacogenomic and Crispr Screens." Patterns 1, no. 5 (2020).
10. Yang, Chi-Hwa, Hsiao-Chin Chou, Yu-Ning Fu, Chi-Ling Yeh, Hui-Wen Cheng, Il-Chi Chang, Ko-Jiunn Liu, Gee-Chen Chang, Ting-Fen Tsai, Shih-Feng Tsai, Hui-Ping Liu, Yi-Cheng Wu, Ya-Ting Chen, Shiu-Feng Huang, and Yi-Rong Chen. "Egfr over-Expression in Non-Small Cell Lung Cancers Harboring Egfr Mutations Is Associated with Marked Down-Regulation of Cd82." Biochimica et Biophysica Acta (BBA) - Molecular Basis of Disease 1852, no. 7 (2015): 1540-49.
11. Fregni, M., Y. Ciribilli, and J. E. Zawacka-Pankau. "The Therapeutic Potential of the Restoration of the P53 Protein Family Members in the -Mutated Lung Cancer." International Journal of Molecular Sciences 23, no. 13 (2022).
12. Zhou, Yaodong, Dongdong Guo, and Yixin Zhang. "Association of Microrna-21 with P53 at Mutant Sites R175h and R248q, Clinicopathological Features, and Prognosis of Nsclc." Molecular Therapy - Oncolytics 19 (2020): 208-17.

13. Hu, Kuan, Yu Xia, Tong Yuan, Yan Lin, and Jin Yang. "Pharmacodynamic-Driven Sequence-Dependent Synergy Effects in Pemetrexed-Osimertinib Combination against Non-Small Cell Lung Cancer (Nsclc): Optimizing Synergy through Sequential Interval." Pharmaceutics 17, no. 8 (2025).

Disclaimer/Publisher's Note: The statements, opinions and data contained in all publications are solely those of the individual author(s) and contributor(s) and not of MDPI and/or the editor(s). MDPI and/or the editor(s) disclaim responsibility for any injury to people or property resulting from any ideas, methods, instructions or products referred to in the content.
